# The Gut Microbiota in Addiction Biology: A Systematic Review of Substance-Induced Dysbiosis and Gut–Brain Axis Alterations

**DOI:** 10.3390/medsci14030367

**Published:** 2026-07-02

**Authors:** Juan Esparza-Sánchez, Diego Alejandro Garibi-Miranda, Marco Chávez-Tinoco, Jorge L. Mejía-Méndez, Maricruz Sepulveda-Villegas, Gildardo Sanchez-Ante, Angélica Lizeth Sánchez-López

**Affiliations:** 1 Escuela de Ingeniería y Ciencias, Tecnológico de Monterrey, Av. General Ramón Corona 2514, Zapopan 45138, Mexico; juan.e.s@tec.mx (J.E.-S.); a01642825@tec.mx (D.A.G.-M.); m_sepulveda@tec.mx (M.S.-V.); 2 Departamento de Genética del Desarrollo y Fisiología Molecular, Instituto de Biotecnología (IBT), Universidad Nacional Autónoma de México (UNAM), Cuernavaca 62210, Mexico; marco.chavez@cinvestav.mx; 3 Escuela de Ingeniería y Ciencias, Tecnológico de Monterrey, Epigmenio González 500, San Pablo, Santiago de Querétaro 76130, Mexico; mejia.jorge@tec.mx

**Keywords:** gut microbiome, substance use disorder(s), short-chain fatty acids, gut–brain axis, intestinal permeability

## Abstract

Background: Growing evidence suggests that chronic substance use disrupts the gut microbiota composition and function, which can contribute to intestinal dysfunction, systemic inflammation, and gut–brain axis dysregulation. However, current evidence remains fragmented and heterogenous, with most studies focusing on individual substances rather than substance-specific microbial signatures. Objective: Therefore, this systematic review synthesizes recent evidence (2019–2025) to characterize the impact of chronic substance use, including alcohol, nicotine, opioids, cocaine, and methamphetamine, on the gut microbiota composition and functional integrity. Methods: Following the PRISMA 2020 guidelines, a total of 91,421 records were identified before screening through searches conducted across electronic databases and publisher platforms, including PubMed, Web of Science, ProQuest, and BSCOhost, among others. After duplication removal and application of the predefined eligibility criteria, 60 studies were selected for qualitative analysis. Results: The findings revealed an interspecies similarity in which chronic substance exposure generally induced dysbiosis characterized by a depletion of beneficial short-chain fatty acid (SCFA)-producing taxa, such as *Lactobacillus*, *Akkermansia*, and *Faecalibacterium*, alongside the enrichment of opportunistic pathogens such as *Escherichia-Shigella*. Alcohol emerged as a particularly potent ecological driver, consistently reducing the richness and diversity of the microbial community. Mechanistically, these alterations are linked to impaired intestinal barrier function, increased lipopolysaccharide translocation, and the activation of systemic inflammatory pathways. Furthermore, substance-specific metabolic fingerprints were identified, including disruptions in glutamate pathways for cocaine and trimethylamine N-oxide precursors for methamphetamine. Preclinical evidence from fecal microbiota transplantation and germ-free models suggests that these microbial shifts actively modulate reward sensitivity and neuroplasticity through the gut–brain axis. Conclusion: Collectively, the data presented in this study support a shift from reductionist addiction models toward a systems-level framework, positioning the gut microbiome as a pivotal, modifiable component of addiction biology and a promising target for novel therapeutic interventions.

## 1. Introduction

Substance use disorders (SUDs) remain a significant global concern, with a higher prevalence in America and Europe. Alcohol, cannabis, opioids, amphetamines, and cocaine are the most commonly used psychoactive substances [1,2], affecting millions of individuals and posing substantial social, economic, and health challenges. Recent evidence from randomized controlled trials, translational studies in patients with addiction-related phenotypes, and preclinical animal models has highlighted the increasing prevalence and complexity of SUDs, driven by factors such as the opioid crisis, the rise of novel psychoactive substances, and the impact of the COVID-19 pandemic on mental health and substance abuse patterns [3,4]. Furthermore, stigma toward people suffering from any addiction or substance use disorder is prevalent, often resulting in diminished medical and psychiatric attention. This neglect can lead to an increase in cases related to substance use disorders or the exacerbation of existing conditions. Studies indicate that SUDs often co-occur with anxiety, depression, bipolar disorder, cognitive deficits, antisocial behavior, and impaired sleep, among others, leading to poorer treatment outcomes and higher relapse rates [5].

The gut microbiota is a dense, metabolically active ecosystem comprising trillions of microorganisms that inhabit the human gastrointestinal tract. These microbial communities function as a dynamic organ that co-evolves with the host and exerts profound effects on physiology, immunity, and metabolism [6,7]. Ecologically, the gut microbiota is characterized by high inter-individual variability and remarkable functional redundancy [8]. Although the taxonomic composition differs substantially between individuals, pathways, such as short-chain fatty acid (SCFA) production, bile acid transformation, and amino acid metabolism, are conserved across healthy populations [9,10]. Microbial metabolites mediate critical host–microbe interactions by regulating epithelial barrier integrity, modulating mucosal and systemic immune responses, and influencing neuroendocrine signaling pathways.

The bidirectional communication between the gut microbiota and host systems, including the immune, metabolic, and nervous systems, underscores its systemic relevance and provides a mechanistic framework for understanding its involvement in diverse pathological conditions. Emerging research underscores the gut–brain axis, a bidirectional communication pathway between the gut and the brain through the vagus nerve, which connects the gastrointestinal tract and its interactions with the gut microbiota to the central nervous system (CNS), both its sympathetic and parasympathetic regions, the enteric nervous system, and the neuroendocrine and neuroimmune systems [11]. Dysbiosis, an imbalance in the gut microbiota, has been associated with numerous health issues. It can increase gut permeability, allowing bacteria or their derivatives to enter the bloodstream. This process triggers an inflammatory response in both the periphery and the CNS, which has been linked to SUDs [12] and related psychiatric conditions [13].

Despite the growing interest in the microbiota–addiction interface, the current evidence remains fragmented and heterogeneous, with studies often focusing on individual substances or specific experimental models. Importantly, the available literature includes both preclinical animal studies and human clinical studies, which differ in their ability to capture chronic substance-induced changes in the gut microbiota composition and functional integrity. Because these models are not directly interchangeable, this review considers them separately when appropriate to better contextualize the evidence. Notably, the evidence base is predominantly preclinical, with most available studies conducted in animal models, while human clinical data remain comparatively limited; however, they have typically focused on a narrower range of substances, specific mechanistic pathways, or older literature [14,15,16,17]. In contrast, the present systematic review provides an updated synthesis of studies published between 2019 and 2025, integrates both preclinical and clinical evidence, and comparatively evaluates multiple commonly abused substances, including alcohol, nicotine, opioids, cocaine, and methamphetamine. By examining shared and substance-specific microbiome signatures and their mechanistic links to SCFA depletion, intestinal barrier disruption, systemic inflammation, and gut–brain axis dysfunction, this review offers a broader and more current perspective on the field. Following the PRISMA 2020 guidelines, this review aims to synthesize and critically evaluate recent evidence on the effects of chronic exposure to these substances on the gut microbiota composition and function.

## 2. Materials and Methods

The search strategy for this systematic review was developed in accordance with the PRISMA 2020 guidelines to ensure a comprehensive, transparent, and reproducible identification of relevant studies [18]. A systematic literature search was conducted across the following search interfaces/publisher platforms: PubMed (n = 17), ScienceDirect (n = 7308), Taylor & Francis (n = 1325), ProQuest (n = 2672), Springer Nature Link (n = 26,794), Web of Science (n = 201), Cambridge Core (n = 52,487), Wiley Online Library (n = 614), and MDPI (n = 3), yielding a combined total of n = 91,421 records prior to exclusion criteria. EBSCOhost returned zero results for the applied search string and was therefore excluded from the record count. The search included studies published between January 2019 and February 2025 to provide an up-to-date synthesis of contemporary animal and human research rather than a historical review. In the following sections, these studies are categorized as preclinical and clinical, respectively.

A keyword-based search strategy was applied consistently across all platforms using Boolean operators. The core search string was ((gut microbiota OR gut microbiome) AND (substance use OR substance abuse)) AND disorder. Where allowed, the search was limited to Title/Abstract fields; otherwise, the full keyword search function was used. To improve retrieval consistency, the same conceptual terms were adapted to the syntax requirements of each platform, and database-specific filters were applied according to platform availability.

For PubMed, the search was restricted to records with available abstracts and filtered by article type, including clinical trials and randomized controlled trials. ScienceDirect was filtered by article type, including research articles and case reports. Taylor & Francis was restricted to articles. ProQuest was filtered by source type (scholarly journals) and document type (case studies and articles). Springer Nature Link was filtered by content type (research articles). The Web of Science was restricted to document type (articles). Cambridge Core was filtered by content type (articles). Wiley Online Library was searched using the keyword strategy followed by an open access filter. MDPI requires only keyword search without additional filters.

**Figure 1:**
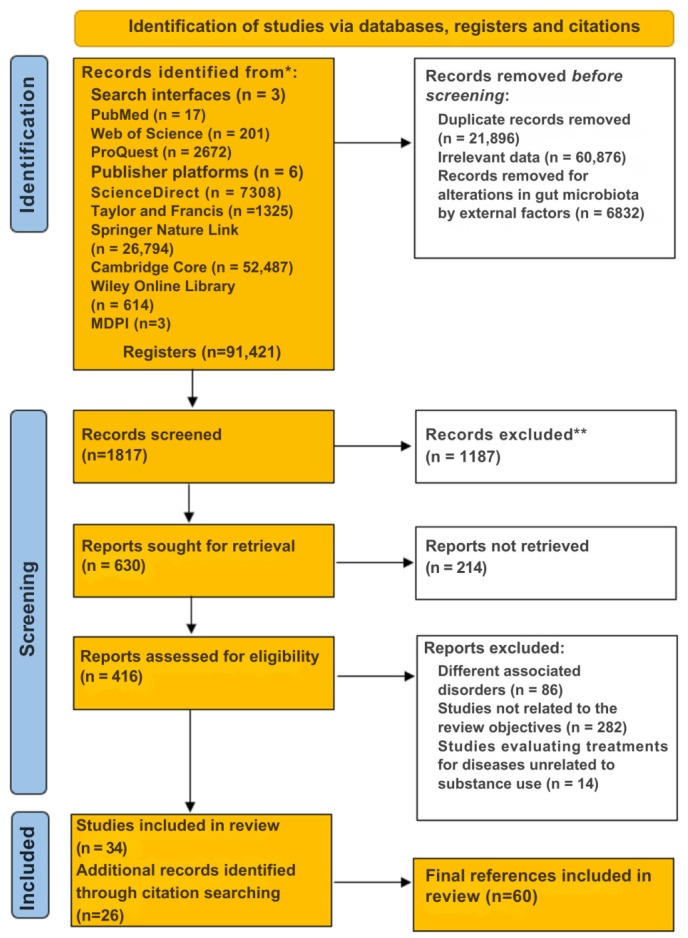
PRIMA 2020 flow diagram illustrating the study selection process. From 91,421 records initially identified, 416 full-text were evaluated for elegibility, and 60 studies were finally included in this systematic review.

Eligibility criteria included original, open-access, peer-reviewed research articles published in English involving either human participants or animal models exposed to substances of abuse and reporting outcomes related to the gut microbiota composition, diversity, or functional alterations. Studies were excluded if they were review articles, editorials, conference abstracts, commentaries, or lacked sufficient methodological detail or microbiota-related outcomes. Studies evaluating disorders unrelated to substance use or interventions focused on unrelated diseases were also excluded. Following database retrieval, we manually identified and removed duplicate records by comparing titles, authors, journals, and publication years across all exported lists, and we excluded studies in which gut microbiota alterations were driven by external, non-substance-related factors prior to screening. Full-text eligibility assessment was independently conducted by two reviewers (J.E.-S. and D.A.G-M.) for all potentially relevant studies using predefined inclusion and exclusion criteria. Any disagreements were resolved through discussion and consensus. Additional references identified through citation searching were also evaluated for eligibility. The complete study selection process is summarized in the PRISMA 2020 flow diagram presented in Figure 1.

**Figure 2:**
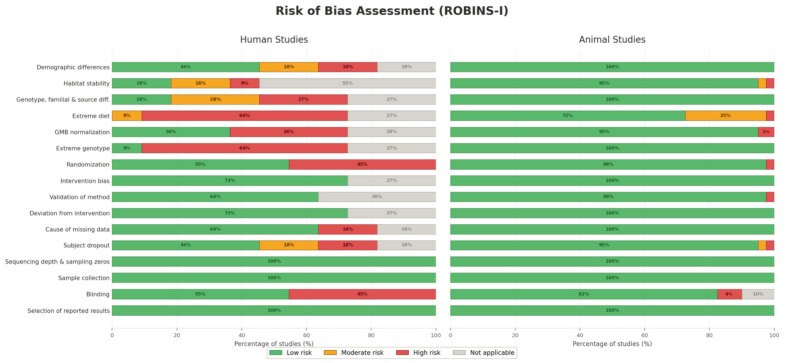
Risk of bias assessment of included studies using ROBINS-I criteria.

Keywords were entered individually into the designated search fields of each database to ensure maximum retrieval as well as accuracy and consistency. The risk of bias in the included studies was evaluated using an adapted version of the Risk of Bias in Non-randomized Studies of Interventions (ROBINS-I) tool, modified to address methodological considerations specific to microbiome research [19]. In this adaptation, we evaluated each included study across a set of domains tailored to both clinical and microbiome-specific sources of bias. These domains comprised demographic differences, habitat stability, genotype, familial and source differences, extreme diet, gut microbiota (GMB) normalization procedures, extreme genotype, randomization, intervention bias, validation of microbiome and clinical methods, deviation from intervention, cause of missing data, subject dropout, sequencing depth, and handling of sampling zeros, sample collection, blinding, and selection of reported results. Each domain was rated as low, moderate, or high risk of bias according to predefined criteria, and these ratings were combined to derive an overall ROBINS judgment for each study, as summarized in Figure 2 and detailed in Supplementary Tables S13 (animal) and S14 (human). Data extraction was performed using standardized tables that included study characteristics, sample type, analytical approach, functional implications, microbiota-related outcomes, and statistical methodologies. When available, information regarding alpha and beta diversity, differentially abundant taxa, microbiota–host interactions, substance use disorder severity, and sex distribution was also extracted and summarized in the Supplementary Materials.

Due to significant methodological and biological differences among the included studies, a meta-analysis was not conducted. There was considerable variation in experimental models, sequencing platforms, bioinformatic pipelines, microbial outcome measures, and reported taxonomic indices, which precluded meaningful quantitative synthesis. In addition, many studies relied on animal models, particularly mice and rats, which limits the direct applicability to human populations. The relatively small number of eligible studies also reduced the statistical robustness required for meta-analytic procedures. Accordingly, the review used a narrative synthesis approach, and no quantitative effect measures were calculated or pooled across studies. The results are descriptive and are organized by substance class and level of evidence (preclinical/clinical). Subgroup analyses were not formally conducted because the number of studies within each substance class and model type was insufficient for meaningful quantitative stratification.

## 3. Results

The results of the risk of bias assessment conducted using the adapted ROBINS-I tool are presented in Figure 2. Staked bar charts summarize the proportions of studies classified as having low (green), moderate (orange), high (red), or not applicable (gray) risk of bias across the predefined methodological domains. The results are presented separately for clinal (left panel) and preclinical studies (right panel).

Overall, preclinical studies were mainly classified as low risk of bias across the evaluated domains. In contrast, clinical studies showed a higher proportion of moderate- and high-risk ratings, particularly in extreme diet, genotype, randomization, blinding, and habitat stability domains. Low-risk ratings were more frequently observed in domains associated with sequencing procedures and sample handling in both study types.

### 3.1. Alcohol

Across clinical and preclinical studies, several consistent patterns emerge despite the differences in experimental design, host species, and analytical approaches. Alcohol exposure consistently induces gut microbial dysbiosis characterized by reduced microbial richness, altered community structure, depletion of beneficial commensals, expansion of generalist or opportunistic taxa, and strong associations with inflammatory and metabolic dysfunction along the gut–liver–brain axis.

In preclinical studies using murine models, amplicon-based sequencing (primarily 16S rRNA) of fecal samples has demonstrated reproducible alcohol-driven shifts in bacterial composition, with reports of reductions in beneficial taxa such as *Lactobacillus* and Cyanobacteria, alongside enrichment of Firmicutes and opportunistic genera including *Escherichia-Shigella* [20,21].

Similar patterns have been observed in rats exposed to alcohol. Different studies documented reductions in beneficial genera such as *Prevotella* and *Akkermansia*, alongside the enrichment of potentially harmful taxa including *Clostridium* [22,23]. Fecal findings were corroborated through cecal and colonic content analyses. Additionally, reductions in richness and the depletion of beneficial taxa such as *Lactobacillus* and *Faecalibaculum rodentium*, accompanied by increases in generalist or pathogen-associated genera, including *Helicobacter*, have been reported [24,25].

Fungal analyses (ITS sequencing) revealed relatively stable fungal α-diversity but reduced bacterial–fungal interaction networks and an increased prevalence of non-*Saccharomyces* yeasts such as *Pichia kudriavzevii*, suggesting disruption of the cross-kingdom ecological balance [25]. More recent studies demonstrated consistent reductions in α-diversity (Chao1, Shannon) and clear β-diversity separation in principal coordinates analysis (PCoA), underscoring alcohol as a dominant ecological force shaping the gut microbial structure [26,27].

In clinical studies, fecal sampling remains the predominant approach due to accessibility and its relevance to gut–brain axis research. In a study enrolling treatment-seeking alcohol use disorder (AUD) participants [28], a reduced microbial diversity with the enrichment of Proteobacteria and depletion of commensals was observed. Similarly, increases in generalist taxa such as *Eisenbergiella*, Firmicutes, and Proteobacteria, alongside reductions in *Faecalibacterium*, *Akkermansia*, and *Lactobacillus*, have been documented [29,30]. The diversity indices most consistently affected included Chao1 and ACE—metrics that are sensitive to richness and rare taxon detection indicating contraction of the microbial population breadth following alcohol exposure. Although most studies relied on amplicon sequencing (16S/ITS), shotgun metagenomics and genetic inference approaches reinforce similar conclusions. It has been demonstrated [31] that altered gut–brain modules link to glutamate and GABA pathways in AUD. In addition, using Mendelian randomization in the FinnGen cohort suggested protective associations for butyrate-producing taxa such as *Eubacterium*, supporting a functional role of SCFA-producing bacteria in alcohol-related phenotypes [31].

Taken together, the findings from murine, rat, and human cohorts suggest that chronic alcohol exposure is frequently associated with reduced gut microbial richness and marked changes in community structure, including shifts in α-diversity indices and consistent β-diversity separation in ordination analyses such as PCoA [20,21,31]. Despite the heterogeneity in specific bacterial and fungal taxa, multiple studies report alterations in bile acid metabolism, intestinal barrier function, and inflammatory signaling pathways, which may reflect overlapping functional consequences of alcohol-related dysbiosis [32,33,34]. Overall, the available evidence is consistent with a pattern of depletion of several commensal or SCFA-producing taxa alongside the expansion of generalist or opportunistic microbes across host species, supporting further investigation of functional microbial signatures and ecological biomarkers, rather than single-taxon associations, as potentially more robust indicators of alcohol-associated gut dysbiosis.

### 3.2. Nicotine

The effects of tobacco and nicotine on the microbial integrity of consumers have been explored using amplicon metagenomics. As with the consumption of other substances, the exploration has been focused on fecal samples [35].

Preclinical studies using rodents to investigate the effects of exposure to cigarette smoke and to chronic nicotine administration showed important differences relative to the controls. One of the most important differences is observed in the reduction in beneficial bacteria to the host gut, such as mucus-producing bacteria and *Lactobacillus*. These reductions are also correlated with generalist and opportunistic bacteria [35,36]. In the case of diversity, effects on reductions in richness and diversity have also been reported [36], along with effects on the response of the host, such as a reduction in the mucus layer, which can also increase host–microbe negative interactions, principally through pathogenic organisms, leading to gut–brain axis disruption [35,36].

In clinical studies comparing smokers vs non-smokers with GWAS data, the presence of *Intestinimonas* and the reductions in *Bifidobacterium* and *Actinobacteria* may reflect a host imbalance, as was also observed with metabolite disruption, particularly with tryptophan/tyrosine, which has an important effect on the gut–brain axis via neurotransmitter metabolites [37]. The findings indicate that exposure to tobacco and nicotine may disrupt microbial integrity primarily by diminishing beneficial gut bacteria while promoting the proliferation of generalist and opportunistic ones. This microbial imbalance appears to be associated with reduced richness and diversity within the gut microbiome, thus provoking a reduced protective mucus layer, which could exacerbate adverse host–microbe interactions, particularly with pathogenic organisms, and contribute to disruptions in the gut–brain axis.

### 3.3. Opioids

Across preclinical and clinical studies, opioid exposure consistently induced gut dysbiosis characterized by reduced commensal Firmicutes and SCFA-producing taxa, increased mucin degraders or opportunistic organisms, and impaired barrier integrity.

A mixed-substance (morphine/fentanyl) preclinical study in rats reported a decreased diversity of Firmicutes and *Bifidobacterium pseudolongum* with an increase in *Akkermansia muciniphila*, accompanied by reduced antimicrobial peptide (Reg3 expression), increased intestinal permeability, and bacterial translocation, indicating opioid receptor–mediated suppression of mucosal immunity [38]. Iriah et al. (2025) showed that germ-free female mice exhibited reduced oxycodone reward behavior compared to wild-type controls, supporting a causal microbiome contribution to opioid reward circuitry and brain connectivity [39].

Anther study reported that morphine self-administration in rats reduced Firmicutes (including *Clostridium* and *Ruminococcus 1*) and decreased the α-diversity (in acute and chronic phases), with associated reductions in SCFA production and correlations with white matter microstructural changes in reward-relevant brain regions [40]. In a clinical study, participants exhibited an increase in Actinobacteria and a decrease in *Faecalibacterium*. A mixed substance clinical study reported decreased diversity in both morphine and opioid human users [41]. Alterations in SCFAs and gut–brain axis disruption have been implicated in addiction-related behaviors and psychiatric symptoms. Baslam et al.’s [42] study showed that chronic fentanyl exposure reduced the microbial density and diversity and decreased beneficial taxa (e.g., *Ligilactobacillus*), concomitant with oxidative stress (higher MDA, lower CAT/SOD) and anxiety and depressive-like behaviors. These findings indicate that gut dysbiosis, characterized by decreases in beneficial commensals such as Firmicutes and SCFA-producing taxa and increases in mucin-degrading or opportunistic bacteria, correlates with impaired intestinal barrier integrity, reduced antimicrobial peptide expression, and increased intestinal permeability, leading to a mixed-substance study reporting decreased diversity in both morphine-CPP mice and human opioid users [43]. In mice, increases in Firmicutes and the depletion of *Lactobacillus* were observed. In a clinical study, participants exhibited an increase in Actinobacteria and a decrease in *Faecalibacterium*. Alterations in SCFAs and gut–brain axis disruption have been implicated in addiction-related behaviors and psychiatric symptoms. In the study by Baslam et al. [42], it was shown that chronic fentanyl exposure reduced microbial density and diversity and decreased beneficial taxa (e.g., *Ligilactobacillus*), concomitant with oxidative stress (higher MDA, lower CAT/SOD) and anxiety and depressive-like behaviors.

### 3.4. Cocaine

Cocaine exposure produces reproducible β-diversity shifts, inflammatory signatures, and alterations in SCFA metabolism, although α-diversity changes are less consistent across models. A preclinical study reported that mice acquiring cocaine self-administration exhibited enrichment of *Barnesiella*, *Ruminococcus*, and *Robinsoniella*, with glutamate metabolism gene enrichment [44]. While α-diversity did not differ, β-diversity separated the acquisition phenotypes, suggesting functional (rather than taxonomic) microbiome signatures associated with cocaine vulnerability. An increased α-diversity (Shannon, observed features) was observed following cocaine exposure and persistent dysbiosis after cessation [45]. In another study [46], repeated cocaine exposure in female mice was found to increase *Akkermansia muciniphila* and to reduce SCFA-producing taxa (*Muribaculaceae* and *Flintibacter*), with significant β-diversity separation despite the unchanged α-diversity. Functional predictions suggested enhanced nucleotide/cofactor metabolism and possible barrier compromise. Clinical evidence showed reduced fecal and oral α-diversity in human cocaine use disorder (CUD) patients, with depletion of butyrate and reductions in *Christensenellaceae*/*Lachnospiraceae*, alongside enrichment of inflammatory taxa (e.g., *Erysipelotrichaceae*) [47]. Functional predictions implicated inflammatory and neurotransmitter synthesis pathways. Additionally, antibiotic-induced SCFA depletion (acetate, propionate, butyrate; increase in Proteobacteria) was shown to alter cocaine motivation and nucleus accumbens transcription [48]. SCFA repletion reversed the behavioral effects, providing causal evidence for SCFA-mediated modulation of cocaine reinforcement.

### 3.5. Methamphetamine

Methamphetamine (METH) exposure consistently induced pronounced and reproducible gut dysbiosis across both preclinical and clinical studies, frequently characterized by alterations in microbial diversity, an increase in the *Firmicutes*/*Bacteroidetes* ratio, enrichment of opportunistic pathogens, impairment of intestinal barrier integrity, oxidative stress, and activation of neuroinflammatory signaling pathways [49]. Collectively, these findings suggest that METH-associated microbial alterations are closely linked to systemic inflammation, neurobehavioral dysfunction, and metabolic disturbances relevant to substance use disorders.

Preclinical data showed that an increased α-diversity, accompanied by an elevated Firmicutes/Bacteroidetes ratio, was linked to cardiotoxicity through activation of the p53 and PI3K/Akt signaling pathways, while fecal microbiota transplantation (FMT) transferred susceptibility to cardiac injury to healthy recipient mice, supporting a causal role of the microbiota [49]. Marked reductions in microbial diversity and depletion of hydrogen- and butyrate-producing bacteria, including *Bacteroidetes* and *Roseburia*, together with enrichment of *Escherichia-Shigella*, were also observed and correlated with neuropsychiatric symptom severity [50]. Prenatal METH exposure induced microbial alterations in mouse offspring characterized by increased Firmicutes and *Verrucomicrobia* and decreased Bacteroidetes, which were associated with oxidative stress and hepatotoxicity [51]. Furthermore, increased *Lachnospiraceae_NK4A136_group* and reduced Muribaculaceae, linking dysbiosis to gut barrier damage, LPS translocation, and PI3K-Akt activation, have been reported [52]. Similarly, an increased *Klebsiella oxytoca* abundance and decreased Bacteroidales abundance correlated with microglial activation and dopaminergic dysfunction in rats [53]. Other findings included elevated Firmicutes, reduced SCFAs, impaired tight junction proteins (ZO-1 and occludin), and activation of TLR4/NF-κB signaling; moreover, SCFA alterations were additionally associated with the SIGMAR1/BDNF/TRKB pathways, suggesting microbial modulation of neuroplasticity [54]. It was also shown that the *Lactobacillus* abundance inversely correlated with MA intake in rats and monkeys, suggesting microbiota-dependent modulation of drug consumption [55].

Clinical studies also identified significant microbial alterations in users. Liu et al. (2024) demonstrated reduced α-diversity in MA abstainers (<3 months), increased opportunistic pathogens, and mediation effects between the microbiota (specifically, *Faecalitalea*) and impulsivity-related behaviors [56]. Furthermore, patients with MUD exhibiting sleep disturbances showed enrichment of inflammatory taxa such as *Escherichia-Shigella*, linking dysbiosis to inflammation and functional impairment [57].

### 3.6. Other Psychotropic Drugs

In the case of less commonly studied psychotropic drugs, evidence on benzodiazepines remains scarce and insufficient to delineate a complete cascade of microbiota-mediated host responses. Nevertheless, an experimental study in zebrafish exposed to benzodiazepines reported marked gastrointestinal alterations, with 16S rRNA amplicon sequencing revealing the proliferation of generalist taxa (*Proteobacteria*, *Firmicutes*, *Bacteroidetes*) and a significant increase in some bacteria with pathogenic potential, as in the case of *Paracoccus* and *Gemmobacter*. Overall decreases in richness and a clear separation of bacterial communities indicated that benzodiazepine exposure reshapes gut ecology, accompanied by disrupted nucleotide and amino acid metabolism, inflammation, oxidative stress, and neurotoxicity in the host [58]. These alterations may also affect neurotransmitter-related pathways and contribute to longer-term neurobiological changes mediated by the gut microbiota.

In mammals, exposure to ketamine, a dissociative anesthetic, has similarly been associated with pronounced shifts in the gut microbial composition. In rats, ketamine administration altered the abundances of *Blastococcus* and *Alcaligenes* and increased other taxa, including *Akkermansia*, *Brachyspira*, and *Desulfovibrio*, with bacterial communities clustering according to drug exposure and indicating strong ecological selection by the drug. According to the retrieved evidence, it was noted that there are different factors that can contribute to the downregulation of *Akkermansia* spp., which has been documented as a promising therapeutic probiotic against metabolic disorders such as obesity, type 2 diabetes, and cardiovascular diseases [59]. Together with this information, species such as *A. muciniphila* have been referred to as beneficial for enhancing intestinal barrier integrity, regulating immune responses, and reducing inflammation [60]. The same species has been reported to enhance the sensitivity to insulin in overweight volunteers during a clinical trial [61]. These compositional changes were linked to altered host–microbe interactions, hippocampal dysfunction, and intestinal barrier disruption, supporting a role for ketamine-induced dysbiosis in microbiota–gut–brain axis perturbation [41].

Data from a clinical study employing 16S rRNA amplicon sequencing of fecal samples showed that individuals with SUDs (methamphetamine/opioids) exhibited reduced gut microbiota diversity and distinct microbial community clustering compared with non-users. This dysbiosis was characterized by a reduction in the abundances of *Faecalibacterium* and *Blautia* communities, and increases in the abundances of pathogens such as *Prevotella* and generalists including *Bacteroidetes* were observed, alongside perturbations in central metabolic pathways, including the citrate cycle and the shikimate pathway, highlighting the multifaceted impact of these drugs on the human holobiont [43].

## 4. Discussion

**Figure 3:**
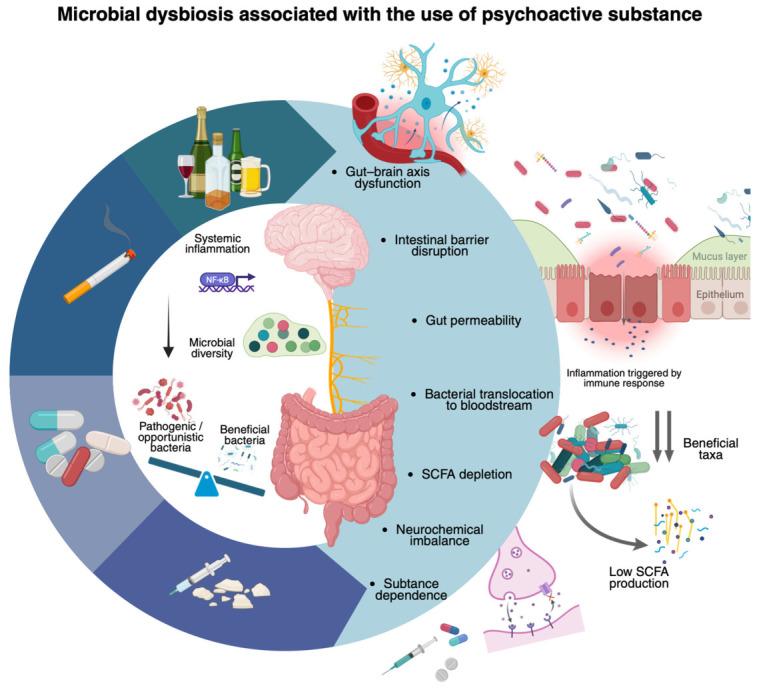
Illustration of the microbial dysbiosis associated with the use of psychoactive substances. Created in BioRender. Sánchez, A. (2026) https://BioRender.com/vu26hg6 (accessed on 28 June 2026).

This systematic review identified a convergent pattern across substances of abuse in which chronic exposure induces reproducible gut microbial dysbiosis characterized by the depletion of SCFA-producing taxa, enrichment of opportunistic microbiota such as *Proteobacteria*, and consistent activation of host inflammatory pathways (Figure 3). Across preclinical and clinical evidence, these microbial alterations are associated with the disruption of epithelial integrity, immune activation, and modulation of neurobehavioral processes relevant to SUDs.

The adapted ROBINS-I assessment indicated that animal studies generally exhibited a low risk of bias across most domains, reflecting the controlled conditions and strong internal validity of preclinical models [20,36,45,62]. In contrast, human studies showed greater methodological variability and higher proportions of moderate to high risk of bias, likely related to population heterogeneity, genetic diversity, sample size limitations, and intervention adherence [28,30,47,50]. Despite these differences, the findings from murine, rat, zebrafish, and human studies consistently support an active role of microbial dysbiosis within the gut–liver–brain axis. Importantly, mechanistic evidence from fecal microbiota transplantation, germ-free models, and SCFA supplementation suggests that microbial metabolites may directly influence addiction-related phenotypes, supporting a functional contribution of the gut microbiota beyond simple association.

The consistency across studies for alcohol is striking. In murine models, reductions in *Lactobacillus* and enrichment of *Escherichia-Shigella* and *Firmicutes* members were accompanied by decreased α-diversity and clear β-diversity clustering in principal coordinates analysis (PCoA) and non-metric multidimensional scaling (NMDS) [20,21,62]. Across studies, ethanol exposure was consistently associated with decreased richness and altered Firmicutes/Bacteroidetes ratios. In one study, NMDS revealed distinct clustering of ethanol-exposed mice relative to controls [63]. Functionally, these microbial shifts correlated with inflammatory and metabolic dysregulation, including NF-κB activation, disruption of secondary bile acid metabolism, and impaired SCFA production [32,34]. Although taxonomic diversity indices were not always significantly altered, β-diversity analyses consistently demonstrated alcohol-driven community restructuring (confirmed by PERMANOVA and related methods), and these compositional changes were associated with host metabolic dysfunction, oxidative stress, and inflammatory activation [31,34,64].

Rat studies reinforced this pattern, showing reductions in *Akkermansia* and *Prevotella* with enrichment of *Clostridium* and associations with oxidative stress and metabolic dysfunction [23,52]. Importantly, cecal and colon analyses extended these observations to barrier impairment, hepatic lipid accumulation, and Th17/Treg imbalance, linking dysbiosis to systemic inflammatory consequences [26,27]. Human data mirrored the preclinical findings, with reduced richness (Chao1/ACE), enrichment of *Proteobacteria*, and depletion of *Faecalibacterium* and *Akkermansia* in AUD populations [28,29,30]. The β-diversity PCoA plots consistently showed clustering of alcohol-exposed individuals separate from controls [31,34,64], and dysbiosis was associated with gut–liver axis disruption, increased intestinal permeability, reduced microbial interaction networks, and neuroplasticity- and inflammation-related gene expression. Furthermore, shotgun metagenomics demonstrated altered gut–brain modules involving glutamate and GABA metabolism, while Mendelian randomization suggested protective associations of butyrate-producing taxa such as Eubacterium [31]. Collectively, these results support a translationally robust model in which the alcohol-induced depletion of SCFA producers drives barrier dysfunction, LPS translocation, immune activation, and neuroinflammatory signaling relevant to addiction and mood dysregulation.

Although fewer in number, the nicotine-related findings demonstrate parallel ecological vulnerability. Rodent studies reported reductions in mucus-associated bacteria and Lactobacillus alongside decreased diversity and mucus layer thinning, a structural change that increases host–microbe contact and inflammatory potential [35,36]. Human GWAS-integrated analyses identified the enrichment of Intestimonas and depletion of *Bifidobacterium* and *Actinobacteria* in smokers, with metabolomic disruption in tryptophan and tyrosine pathways [37]. These metabolic perturbations are particularly relevant to serotonergic and dopaminergic neurotransmission, suggesting that nicotine-associated dysbiosis may amplify neurochemical imbalances implicated in dependence and affective comorbidities. Overall, the available evidence indicates that tobacco and nicotine exposure may disrupt microbial integrity by diminishing beneficial gut bacteria while promoting the proliferation of generalist and opportunistic taxa, a microbial imbalance associated with reduced richness and diversity, a compromised mucus layer, exacerbated adverse host–microbe interactions, and potential disturbances along the gut–brain axis.

Opioid exposure demonstrates a strong barrier-immune signature. In mice, morphine and fentanyl reduced *Firmicutes* and *B. pseudolongum* while increasing *A. muciniphila*, accompanied by reduced Reg3γ expression, increased permeability, and bacterial translocation [38]. These data support the idea that that opioid receptor activation suppresses mucosal immunity, thereby reshaping microbial ecology. Germ-free models further demonstrated attenuated oxycodone reward behavior and altered brain connectivity, implicating microbiota-dependent modulation of the reward circuitry [39]. Morphine-conditioned place preference models revealed reduced *Lactobacillus* and altered SCFA levels, while human opioid users exhibited decreased *Faecalibacterium* and increased *Actinobacteria*, paralleling the animal findings [41]. Brunetti et al. showed that morphine self-administration reduced *Firmicutes* and correlated with white matter microstructural changes in reward-relevant regions [40]. Baslam et al. extended this to fentanyl, linking reduced microbial diversity and *Ligilactobacillus* depletion to oxidative stress and anxiety/depressive-like behaviors [42]. Overall, these data indicate opioid-associated gut dysbiosis characterized by the loss of beneficial commensals and SCFA-producing taxa and enrichment of mucin-degrading or opportunistic bacteria, in association with impaired intestinal barrier integrity, reduced antimicrobial peptide expression, increased permeability and bacterial translocation, and alterations in reward-related neurocircuitry and affective behaviors. Together, these studies suggest a coherent opioid–microbiome–brain axis in which SCFA depletion and epithelial compromise may sensitize neuroimmune pathways and modulate reward sensitivity.

Cocaine studies have emphasized functional metabolic modulation over uniform taxonomic shifts. Tran et al. demonstrated enrichment of *Barnesiella*, *Ruminococcus*, and *Robinsoniella* in mice acquiring cocaine self-administration, with enrichment of glutamate metabolism genes despite the unchanged α-diversity [44]. This suggests that microbial functional capacity, rather than diversity alone, predicts cocaine vulnerability. Gerace et al. identified reduced α-diversity and depletion of butyrate-producing *Christensenellaceae* and *Lachnospiraceae* in cocaine use disorder (CUD) patients, alongside enrichment of inflammatory taxa such as *Erysipelotrichaceae* [47]. Crucially, Meckel et al. provided causal validation: antibiotic-induced SCFA depletion increased cocaine motivation and altered nucleus accumbens transcription [48], while SCFA repletion reversed the behavioral changes. Angoa-Pérez et al. reported increased *A. muciniphila* and reduced *Muribaculaceae* and *Flintibacter* following repeated cocaine exposure, with β-diversity separation and predicted barrier compromise [46]. These data collectively highlight SCFAs as mechanistic mediators linking microbial ecology to mesolimbic dopaminergic plasticity.

METH appears to have a highly systemically disruptive profile. In mice, Liu et al. demonstrated an increase in the Firmicutes/Bacteroidetes ratio and cardiotoxicity transferable by fecal microbiota transplantation, implicating microbiota as a casual contributor in animal models [49]. This key mechanistic study was assessed as having an overall low risk of bias under the modified ROBINS-I tool, given its 100% adherence to demographic/species for matching and robust sequencing depth quality controls. Another study reported minimal compositional changes but identified elevated trimethylamine N-oxide (TMAO) precursors, implicating metabolite-driven neuroinflammation via TLR4/NF-κB signaling, and depressive-like behaviors independent of major taxonomic alterations [62]. Furthermore, there was an association between prenatal altered mouse offspring microbiota and oxidative stress markers, suggesting developmental vulnerability [51]. Human METH users showed reduced diversity and depletion of butyrate producers such as *Roseburia*, with enrichment of Escherichia-Shigella correlating with psychiatric symptom scales [50]. Barrier disruption with reduced tight junction proteins (ZO-1, occludin) and increased LPS mechanistically linked dysbiosis to systemic inflammation [21,65]. Correlations between SCFAs and SIGMAR1/BDNF/TRKB signaling pathways further connect microbial metabolites to neuroplasticity. He et al. demonstrated an inverse relationship between the Lactobacillus abundance and METH intake across species, implying microbiota-dependent modulation of drug consumption behavior [55]. Collectively, these findings position METH-induced dysbiosis at the intersection of barrier failure, oxidative stress, and neuroimmune activation.

However, these results challenge reductionist models of addiction confined to central dopaminergic dysfunction and instead support a systems-level framework in which peripheral microbial ecosystems shape reward sensitivity, stress reactivity, and psychiatric comorbidity.

Despite advancements, variability in sequencing methodologies, a scarcity of longitudinal human data, and confounding lifestyle factors limit the ability to draw definitive conclusions. Many human studies are cross-sectional, hindering the ability to determine whether dysbiosis precedes or follows chronic drug exposure. Additionally, the heterogeneity in participant demographics, environmental exposures, and comorbid conditions further complicates the interpretation of microbiome alterations. The complexity of host–microbiome interactions and the influence of external variables such as diet, medications, and stress necessitate more controlled and comprehensive study designs. Future research should incorporate longitudinal multi-omics profiling alongside neuroimaging and behavioral phenotyping to elucidate the mechanistic pathways from microbial disturbances to neural circuit remodeling. Integrating standardized sequencing protocols and larger, more diverse cohorts will enhance the reproducibility and generalizability. Moreover, experimental models that allow causal inference and intervention studies targeting the microbiome could provide critical insights into the therapeutic potential. Combining these approaches will advance the understanding of the temporal dynamics and functional consequences of microbiome changes in chronic drug exposure and related neuropsychiatric outcomes. However, the evidence consistently suggests that addictive substances alter gut microbial ecosystems in ways that are mechanistically linked to immune activation, metabolic dysfunction, and neuroplastic changes central to the pathophysiology of substance use disorders (SUDs). The convergence of cross-species findings, along with emerging causal experiments, suggests that the gut microbiome is not merely a biomarker but a modifiable component of addiction biology. A logical translational step involves the development of rigorously designed microbiome-targeted interventions aimed at restoring microbial metabolic balance and mitigating the systemic inflammatory and neurobehavioral processes that perpetuate substance dependence.

Although rodent models provide baseline mechanistic insight, cross-species extrapolation in microbiome research should be interpreted with caution. Differences in baseline diet, gastrointestinal anatomy, microbial ecology, and host epigenetic responses may contribute to distinct microbiome responses between animals and humans [66].

Beyond these shared and substance-specific metabolic shifts, the present evidence also supports a role for microbiota-derived neurotransmitter pathways in addiction biology. Alterations in microbial communities may influence host signaling through glutamate, GABA, serotonin, dopamine, and tryptophan/tyrosine metabolism, thereby affecting reward processing, mood regulation, and neuroinflammatory responses [67,68]. In parallel, microbial metabolites such as SCFAs may contribute to epigenetic regulation by modulating histone acetylation, chromatin accessibility, and downstream gene expression programs [69]. Together, these mechanisms provide a plausible functional bridge between substance-induced dysbiosis and persistent neurobehavioral changes [70].

This review has several limitations. First, the included studies showed substantial heterogeneity in models, sequencing platforms, analytic pipelines, and microbiome outcomes, which precluded a meta-analysis and required a narrative synthesis. Second, the evidence base is dominated by preclinical studies, whereas human data are fewer, often cross-sectional, and frequently at moderate to high risk of bias due to incomplete control of diet, co-medications, comorbidities, and socioeconomic factors, limiting causal inference. Third, most studies predominantly utilized 16S/ITS amplicon sequencing, with minimal integration of multiomics approaches, and depended on functional interpretations derived from inferences. Fourth, the review was not prospectively registered in PROSPERO, INPLASY, or any similar registry before study selection or data extraction and did not include a formal certainty-of-evidence or publication-bias assessment. Finally, evidence for nicotine, benzodiazepines, and other psychotropic drugs remains sparse and heterogeneous, so the corresponding microbiota patterns should be considered preliminary.

## 5. Conclusions

Among the substances evaluated, the evidence base is most extensive for alcohol, followed by methamphetamine, opioids, and cocaine, with tobacco/nicotine currently supported by comparatively fewer and more heterogeneous studies. The effects of nicotine on microbial communities and the host response still have some limitations, associated with the lack of an accurate method for measuring exposure and with how researchers can obtain a complete picture of the effects. The evidence supports alcohol as a potent modulator of gut microbial ecology, with downstream inflammatory, metabolic, and neurobiological consequences. The convergence of findings across sequencing platforms and host species underscores the robustness and translational relevance of alcohol-induced gut dysbiosis. Opioid exposure promotes SCFA depletion, epithelial barrier disruption, reduced antimicrobial defenses, oxidative stress, and neurobehavioral alterations. Both reward-related behaviors and brain structural connectivity appear to be microbiota sensitive, supporting a bidirectional opioid–microbiome–brain axis. Cocaine induces community restructuring with consistent SCFA-related perturbations and inflammatory shifts. Functional metabolic pathways, notably glutamate and SCFA metabolism, correlate with cocaine reinforcement and motivational phenotypes. Mechanistically, SCFA depletion emerges as a plausible driver of altered reward neurocircuitry. Methamphetamine-associated alterations induce robust gut barrier dysfunction, SCFA depletion, LPS-mediated systemic inflammation, oxidative stress, and neuroimmune activation. Both cardiotoxic and neuropsychiatric phenotypes appear to be microbiota sensitive, with emerging evidence that specific taxa, such as *Lactobacillus*, may modulate drug intake behavior. Taken together, these findings suggest that microbiota-targeted interventions represent an important but still experimental potential that will require rigorous mechanistic studies and well-designed clinical trials. Future studies should prioritize longitudinal human cohorts, standardized exposure assessments, multi-omics integration, and mechanistic experiments that combine microbiome profiling with neurotransmitter, epigenetic, inflammatory, and behavioral endpoints to clarify causality and identify substance-specific microbial signatures.

## Data Availability

The original contributions presented in this study are included in the article/Supplementary Material. Further inquiries can be directed to the corresponding author.

## Supplementary Materials

The following supporting information can be downloaded at: https://www.mdpi.com/article/10.3390/medsci14030367/s1, Table S1. Summary of alcohol-associated gut microbiota studies; Table S2. Substance-specific alterations in gut microbiota across substance use disorders; Table S3. Nicotine-associated alterations in gut microbiota; Table S4. Opioid-associated alterations in gut microbiota; Table S5. Cocaine-associated alterations in gut microbiota; Table S6. Methamphetamine-associated alterations in gut microbiota; Table S7. Characteristics of included studies evaluating alcohol abuse and microbiota; Table S8. Characteristics of included studies evaluating methamphetamine abuse and microbiota; Table S9. Characteristics of included studies evaluating opioid abuse and microbiota; Table S10. Characteristics of included studies evaluating multiple SUDs and microbiota; Table S11. Characteristics of included studies evaluating cocaine use and microbiota; Table S12. Characteristics of included studies evaluating methamphetamine use and microbiota; Table S13. Risk of bias assessment of included animal studies; Table S14. Risk of bias assessment of included human studies; Figure S1. PRISMA 2020 Checklist; Figure S2. Main taxonomic shifts shared across substances of abuse and specific drugs contributing to the specific patterns.
