# Supplementary material for: The Gut Microbiota in Addiction Biology: A Systematic Review of Substance-Induced Dysbiosis and Gut–Brain Axis Alterations
Source: Med Sci (Basel). 2026 Jul 2;14(3):367. doi: 10.3390/medsci14030367 (PMC13413537; doi:10.3390/medsci14030367)
Supplement: Supplementary file 1 [file medsci-14-00367-s001.zip › medsci-4375612-supplementary.pdf]

Supplementary material

The Gut Microbiota in Addiction Biology: A Systematic Review of Substance-Induced Dysbiosis and Gut–Brain Axis Alterations

Juan Esparza-Sánchez [1], Diego Alejandro Garibi-Miranda [1], Marco Chávez-Tinoco [2], Jorge L. Mejía-Méndez [3], Maricruz Sepulveda-Villegas [1], Gildardo Sanchez-Ante [1,*] and Angélica Lizeth Sánchez-López [1,*]

[1] Tecnológico de Monterrey, Escuela de Ingeniería y Ciencias, Av. General Ramón Corona 2514, Zapopan 45138, Mexico; juan.e.s@tec.mx (J.E.-S.); a01642825@tec.mx (D.A.G.-M.); m_sepulveda@tec.mx (M.S.-V.).

[2] Departamento de Genética del Desarrollo y Fisiología Molecular, Instituto de Biotecnología (IBT), Universidad Nacional Autónoma de México (UNAM), Cuernavaca 62210, Mexico; marco.chavez@cinvestav.mx (M.A.C.-T.).

[3] Tecnológico de Monterrey, Escuela de Ingeniería y Ciencias, Epigmenio González 500, San Pablo, Santiago de Querétaro 76130, Mexico; mejia.jorge@tec.mx (J.L.M.-M.).

* Correspondence: gildardo.sanchez@tec.mx (G.S.-A.); alsl@tec.mx (A.L.S.-L.)

Table S1: Summary of alcohol-associated gut microbiota studies.

Sample Type	Groups	Analytical Approach	Key Microbial Changes	Functional Implications	Alpha Diversity	Beta Diversity	Key Taxa Modulated	Microbiota-Host Interaction	Statistical Approach	Limitations	Study
Mouse fecal samples	Alcohol-exposed (CIAE) vs. Control	16S rDNA sequencing; LC-MS metabolomics	↑ Firmicutes<br>↓ Bacteroidetes<br>↑ Ruminococcaceae<br>↑ Lachnospiraceae<br>↑ Verrucomicrobia<br>↓ Cyanobacteria	Disrupted intestinal barrier/BBB<br>LPS → TLR4 activation → microglial M1 polarization<br>LPS → neuroinflammation<br>Altered SCFA metabolism	N/A	Significant clustering (PCA)	↑ Verrucomicrobia<br>↓ Cyanobacteria<br>↑ Ruminococcaceae<br>↑ Lachnospiraceae	LPS → TLR4 activation → microglial M1 polarization<br>LPS → neuroinflammation<br>Altered SCFA metabolism	Two-way ANOVA; t-tests; PERMANOVA	Limited metabolomic coverage<br>Mouse model	[60]
Human fecal samples	AUD participants; high vs. low reducers	16S rRNA sequencing; LC-MS metabolomics	↓ Faecalibacterium<br>↑ Collinsella<br>↑ Eisenbergiella<br>↓ Roseburia<br>↓ Akkermansia	GABA/tryptophan pathways linked to drinking behavior<br>Neuroactive metabolite shifts<br>Tryptophan metabolites → neurotoxicity	No significant differences	Significant clustering (PERMANOVA, $p = 0.034$)	↑ Collinsella<br>↑ Eisenbergiella<br>↓ Roseburia<br>↓ Akkermansia	Gut GABA ↔ drinking reduction<br>Tryptophan metabolites → neurotoxicity	PERMANOVA; LEfSe; linear regression	Small sample (n=32)<br>No diet/antibiotic data<br>Observational	[10]
Mouse fecal samples	Control, CEE, CEE+LGG, CEE+antibiotics	16S rRNA sequencing (V3–V4)	↓ Lactobacillus<br>↓ Akkermansia<br>↑ Escherichia-Shigella<br>↓ Lachnospiraceae	Systemic inflammation<br>Neuroinflammation<br>Cognitive deficits	↓ Richness;<br>↓ diversity	Significant separation	↓ Beneficial taxa<br>↑ Pathobionts	Gut-brain axis disruption; neuroinflammation	ANOVA; Dunnett post-hoc	Short-term<br>Male-only<br>Limited functional data	[41]
Mouse fecal samples	Control, Ethanol-fed, Ethanol+LH	16S rRNA sequencing (V3–V4)	↓ Lactobacillus<br>↓ Akkermansia<br>↓ Fusobacterium<br>↑ Faecalibaculum<br>↑ Bacteroides	Gut barrier dysfunction<br>Hepatic steatosis<br>↑ LPS/inflammation	↓ Richness; no diversity change	Significant separation	↓ SCFA producers<br>↑ Pro-inflammatory taxa	Gut-liver axis disruption; endotoxemia	Mann–Whitney U; Spearman	Female-only<br>No colonization verification	[33]
Mouse fecal samples	Ethanol-fed vs. pair-fed controls	16S rRNA and ITS sequencing	↓ Faecalibaculum rodentium<br>↑ Helicobacter sp.<br>↑ Pichia kudriavzevii	Gut-liver axis disruption<br>Impaired intestinal barrier<br>Systemic inflammation<br>↓ Lysozyme expression	↓ Bacterial richness (Simpson)<br>No fungal diversity change	Significant shift (PCoA, weighted UniFrac)	↑ Pichia kudriavzevii<br>↑ Helicobacter sp.<br>↓ F. rodentium	NOD2–IL-22–STAT3 pathway activation<br>↓ Lysozyme expression	ANOVA; Mann–Whitney U; LEfSe; Random Forest	Mouse model limitations<br>Fungal role understudied	[57]
Rat fecal samples	UChB-Alcohol, UChB-Water, Wistar control	16S rRNA sequencing; bEV isolation	↑ Proteobacteria<br>↓ Bacteroidetes	bEVs transmit alcohol preference via vagus nerve<br>Independent of systemic inflammation	No significant differences	Significant clustering (PERMANOVA, $p = 0.002$)	↑ Blautia, Ruminococcus<br>↓ Prevotella, Butyricococcus	Vagus nerve mediates bEV-induced alcohol intake	PERMANOVA; Kruskal–Wallis; ANOVA	Rat model limitations<br>bEV cargo not fully characterized	[12]
Human fecal samples	Treatment-seeking AUD individuals	Shotgun metagenomics; Gut-Brain Modules	↑ Proteobacteria<br>↓ commensals (e.g., Lactobacillus)	Altered neuroactive signaling (GABA, glutamate)<br>Associated with AUD symptoms	↓ Diversity (Shannon)	Not reported	↑ Blautia, Escherichia coli<br>↓ Bacteroides, Bifidobacterium	Associations with brain morphometry (amygdala)<br>Links to AUD symptom severity	Pearson correlation; FDR correction	Small sample (n=16)<br>Cross-sectional<br>No healthy controls	[35]
Human fecal samples	Low PEth vs. High PEth	16S rRNA sequencing (V3–V4)	↑ Firmicutes<br>↓ Bacteroidota<br>↓ Verrucomicrobiota<br>↓ Akkermansia<br>↓ Bacteroides<br>↑ Sutterella	Increased Firmicutes/Bacteroidota ratio<br>Reduced Akkermansia → barrier dysfunction<br>↑ Sutterella → inflammation	No significant differences	Trend toward separation (weighted UniFrac, $p = 0.106$)	↓ Akkermansia<br>↓ Bacteroides<br>↑ Sutterella	Reduced barrier function<br>Increased inflammatory signaling	PERMANOVA; ANCOM-BC2; GLM	Small sample size<br>Clinical variability<br>No dietary control	[20]
Mouse fecal samples	Ethanol vs. Control	16S rRNA sequencing (V3–V4)	↑ Prevotellaceae<br>↓ Bacillus	↑ Prevotellaceae associated with inflammation<br>↓ Bacillus may impair gut integrity	N/A	Significant clustering (NMDS)	↑ Prevotellaceae<br>↓ Bacillus	↑ LPS translocation<br>NF-κB and NLRP3 inflammasome activation	One-way ANOVA; Tukey test	Single-dose intervention<br>No chronic alcohol model	[50]
Mouse fecal samples	Control, Ethanol, Ethanol + Adlay	16S rDNA sequencing	↑ Firmicutes<br>↓ Bacteroidota<br>↓ Actinobacteria<br>↓ Proteobacteria	Gut barrier dysfunction<br>Systemic inflammation	↓ Richness (Chao1, ACE)	Distinct clustering (PCoA)	↑ Clostridium_sensu_stricto_1<br>↑ Romboutsia<br>↓ Muribaculaceae	Associated with liver inflammation<br>Oxidative stress	One-way ANOVA; Tukey test	Short-term study<br>Animal model limitations	[7]
Human fecal samples	Control, Alcohol-withdrawn, Lavender oil treatment	16S rDNA sequencing (PacBio)	↓ Bacteroidota<br>↓ Muribaculaceae<br>↑ Monoglobus	Depression-like behavior<br>Inflammation	↓ Richness (Chao1, ACE)	Distinct clustering (PCoA)	↓ Muribaculaceae<br>↑ Monoglobus<br>↓ Lachnospiraceae	Linked to hippocampal inflammation	ANOVA; LEfSe	Small sample size<br>Withdrawal phase only	[26]
Mouse fecal samples	Normal, Alcohol model, Fucoidan treatment	16S rRNA sequencing; targeted metabolomics	↑ Proteobacteria<br>↓ Bacteroides<br>↓ Parabacteroides	Gut-liver axis disruption<br>Inflammation	↓ Richness (Chao1, Shannon)	PCoA separation	↑ Helicobacter<br>↓ Bacteroides<br>↓ Parabacteroides	NF-κB/MAPK activation<br>Altered bile acid metabolism	ANOVA; Tukey test	Limited sample size<br>No human validation	[47]
Mouse fecal samples	Young/aged, ethanol vs. control	16S rRNA sequencing	↑ Eggerthella<br>↑ Holdemania<br>↓ Roseburia<br>↓ Ruminococcaceae	Barrier dysfunction<br>TNFα-driven inflammation	No change in richness;<br>↓ Shannon (aged)	PERMANOVA ($p < 0.001$)	↑ Eggerthella, Holdemania<br>↓ Roseburia	AMP downregulation<br>Gut-liver axis disruption	PERMANOVA; LEfSe; Spearman	Short-term exposure<br>Limited mechanistic validation	[36]
Human fecal samples	AD patients vs. healthy controls	16S rRNA and ITS1 sequencing	**Bacteria:**<br>↑ Lachnospiraceae<br>↑ Fusobacterium<br>↑ Saccharimonadaceae<br>↓ Ruminococcaceae<br>↓ Roseburia<br>↓ Erysipelotrichaceae_UCG-003<br>**Fungi:**<br>↑ Saccharomyces<br>↑ Kurtzmaniella<br>↓ Candida<br>↓ Papillotrema<br>↓ Lodderomyces	Gut dysbiosis<br>Increased intestinal permeability<br>Altered CCK/BDNF signaling<br>Reduced trans-kingdom interactions	↓ Fungal richness (Chao)	Significant separation (PCoA, PERMANOVA)	Bacteria: ↑ Lachnospiraceae, ↑ Fusobacterium, ↓ Ruminococcaceae<br>Fungi: ↑ Saccharomyces, ↓ Candida	Reduced bacteria–fungi interactions<br>↑ CCK; ↓ BDNF	LEfSe; Wilcoxon; PERMANOVA	Small sample<br>Male-only cohort<br>No mechanistic validation	[50]
Rat fecal samples	Control, HFHS + Alcohol, Extract-treated	16S rDNA sequencing; metabolomics	↓ Bacteroidota<br>↑ Firmicutes<br>↓ Akkermansia<br>↑ Clostridium<br>↑ Faecalibaculum	Disrupted lipid metabolism<br>PPARα/γ-mediated inflammation<br>Oxidative stress	↓ Shannon/Simpson	PCoA separation	↑ Firmicutes/Bacteroidota ratio<br>↓ Akkermansia<br>↑ harmful genera	Gut dysbiosis linked to steatosis and inflammation	t-test; ANOVA; LEfSe	Limited mechanistic insight<br>No human validation	[28]
Mouse fecal samples	Pubertal vs. adult; alcohol vs. control	Shotgun metagenomics	**Adults:**<br>↓ Lactobacillus<br>↑ Alistipes, Parabacteroides<br>**Pubertal:**<br>↓ Lactobacillus taiwanensis<br>↑ Bifidobacterium	↓ Bile acid conversion<br>↓ indole metabolism<br>↑ oxidative stress	No change (pubertal); dynamic in adults	Significant in adults (Bray–Curtis)	Adults: ↓ Lactobacillus; ↑ Alistipes<br>Pubertal: ↑ Bifidobacterium	Barrier damage (adults)<br>↑ ALDH (pubertal protection)	ANOVA; LEfSe; DESeq2	Murine model<br>Short exposure	[55]
Mouse fecal samples	Ethanol-fed vs. control	16S rRNA sequencing (V3–V4)	↑ Dubosiella<br>↓ Lachnospiraceae<br>↓ Ruminococcaceae<br>↓ Alistipes	Barrier improvement (intervention)<br>Reduced inflammation<br>Enhanced antioxidant capacity	↓ Diversity (ethanol)	Altered (PCoA)	↑ Dubosiella<br>↑ Bifidobacterium (intervention)	Nrf2/HO-1 activation<br>TLR4/NF-κB suppression	ANOVA; QIIME2	Short experimental duration<br>No human data	[40]
Mouse fecal samples	Binge-on-chronic ethanol vs. control	16S rRNA and ITS sequencing	↑ Dubosiella<br>↑ Faecalibaculum<br>↓ Lachnospiraceae<br>↓ Blautia<br>Fungi: ↑ Eurotium, ↓ Kazachstania	Increased *K. pneumoniae* colonization<br>Reduced secondary bile acids (e.g., lithocholic acid)	↓ Bacterial diversity	Altered (PCoA)	↑ Dubosiella<br>↓ Lachnospiraceae<br>↑ Eurotium (fungi)	Secondary bile acid metabolism disruption	Wilcoxon; LEfSe	Small sample size<br>Limited mechanistic validation	[43]
Mouse fecal samples	Control vs. alcohol	16S rDNA sequencing; SCFA analysis	↑ Desulfovibrio<br>↓ Muribaculaceae<br>↑ Firmicutes/Bacteroidota ratio	↑ LPS → TLR4/NF-κB activation<br>↓ SCFAs → barrier dysfunction	↓ Chao1, Shannon	NMDS: clear separation	↑ Desulfovibrio<br>↓ Akkermansia<br>↓ Muribaculaceae	Gut barrier dysfunction;<br>Th17/Treg imbalance	ANOVA; Tukey test	Murine model<br>Mixed acute/chronic effects	[48]
Mouse fecal samples	Control vs. acute alcohol (AALI)	16S rRNA sequencing; LC-MS	↑ Proteobacteria<br>↑ Escherichia-Shigella<br>↓ Lactobacillus<br>↓ Lachnospiraceae	↓ SCFAs → leaky gut<br>↑ LPS → oxidative stress	↓ Diversity	Distinct clustering (PCoA)	↑ Bacteroides<br>↑ Streptococcus<br>↓ Verrucomicrobiota	Tight junction disruption;<br>CYP2E1-mediated liver injury	Pearson; ANOVA	Acute model only<br>No human validation	[44]
Human fecal/blood samples	AUD vs. controls	16S rRNA sequencing; PICRUSt2	↑ Succinivibrionaceae<br>↓ Lachnospiraceae	SCFA depletion<br>Inflammation and oxidative stress pathways	Non-significant trends	Minimal separation (NMDS)	↑ Succinivibrio<br>↓ Agathobacter	Associations with neuroplasticity genes (BDNF, GRIA1)	edgeR; Spearman; ANOSIM	Small sample (n=20)<br>Sex imbalance<br>No dietary control	[24]
Human (GWAS data)	AUD vs. non-AUD (FinnGen)	Mendelian randomization	Protective: ↑ Eubacterium ventricuum<br>↑ Porphyromonadaceae	Synaptic signaling<br>Tryptophan metabolism	N/A	N/A	↑ Eubacterium (butyrate)<br>↑ Porphyromonadaceae	Causal links to amino acid metabolism (MR)	IVW; MR-Egger; MVMR	Indirect evidence<br>No direct microbiome data	[52]

Table S2: Substance-specific alterations in gut microbiota across substance use disorders.

Substance	Sample Type	Groups	Analytical Approach	Key Microbial Changes	Functional Implications	Alpha Diversity	Beta Diversity	Key Taxa Modulated	Microbiota-Host Interaction	Statistical Approach	Limitations	Study
Methamphetamine / Opioids	Human fecal samples	SUD vs. non-SUD	16S rRNA sequencing; machine learning	↑ Bacteroides<br>↑ Prevotella_9<br>↓ Faecalibacterium<br>↓ Blautia	↑ Citrate cycle activity<br>↓ Shikimate pathway	↓ Diversity (Shannon, Sobs)	PCoA clustering (ANOSIM)	↑ Bacteroides<br>↓ Faecalibacterium	Machine learning links microbiota to SUD (AUC = 0.9)	Wilcoxon; LEfSe; Random Forest	Cross-sectional<br>Regional bias<br>No causality	[32]
Ketamine	Rat fecal, ileum, colon, brain	Ketamine vs. saline control	Metagenomics; MRI; histology	↑ Akkermansia<br>↑ Brachyspira<br>↑ Desulfovibrio<br>↓ Blastococcus<br>↓ Alcaligenes	Hippocampal dysfunction<br>Intestinal barrier disruption	Not reported	Significant clustering (PCoA)	↑ Pathobionts<br>↓ Beneficial taxa	Correlated with CPP score<br>Brain activity (ALFF)<br>Intestinal morphology	Mann–Whitney; t-test; correlation	Animal model<br>No mechanistic validation	[54]
Flunitrazepam	Zebrafish gut	Control vs. FLZ exposure	16S rRNA sequencing; metabolomics	↑ Proteobacteria<br>↑ Actinobacteria<br>↓ Firmicutes<br>↓ Bacteroidota<br>↑ Paracoccus<br>↓ Cetobacterium	Disrupted amino acid/nucleotide metabolism<br>Oxidative stress<br>Neurotoxicity	↓ Richness	Significant clustering (PCA/NMDS)	↑ Pathogenic taxa<br>↓ Beneficial taxa	Gut barrier disruption<br>LPS leakage<br>Neuroinflammation	ANOVA; Spearman	Animal model<br>Short-term exposure	[27]

Table S3: Nicotine-associated alterations in gut microbiota.

Sample Type	Groups	Analytical Approach	Key Microbial Changes (Nicotine vs. Control)	Functional Implications	Alpha Diversity	Beta Diversity	Key Taxa Modulated	Microbiota-Host Interaction	Statistical Approach	Limitations	Study
Plasma / Fecal samples (Mice)	Chronic nicotine vs. saline	ELISA; 16S rRNA sequencing	↓ Lactobacillus<br>↓ Oxytocin (PVN, VTA, CPU)	Reduced nicotine self-administration<br>Gut–brain axis disruption	Not reported	Not reported	↓ Lactobacillus	Oxytocin-mediated regulation of nicotine dependence	Kruskal–Wallis; Dunn's test	No human validation<br>No diversity metrics	[49]
Fecal samples (humans)	Smokers vs. non-smokers	GWAS; Mendelian randomization	↑ Intestinimonas<br>↓ Bifidobacterium<br>↓ Actinobacteria	Positive feedback on smoking behavior<br>Altered tryptophan/tyrosine metabolism	Not reported	Not reported	↑ Intestinimonas<br>↓ Bifidobacterium	Gut–brain axis via neurotransmitter metabolism	IVW; MR-Egger; cML-MA	European ancestry bias<br>No metabolomics<br>No diversity data	[13]
Colon / Fecal samples (mice)	Smoke-exposed vs. air-exposed	16S rRNA sequencing; PNA-FISH	↓ Mucus-associated bacteria<br>↑ Enterobacteriaceae<br>↓ Parabacteroides	Impaired gut barrier<br>Increased systemic and pulmonary inflammation	↓ Shannon (antibiotic-treated)	Significant differences (PERMANOVA)	↑ Enterobacteriaceae<br>↓ Parabacteroides	Reduced mucus layer<br>Increased bacterial encroachment	ANOVA; Tukey; DESeq2	Animal model<br>Short exposure duration	[56]

Table S4: Opioids-associated alterations in gut microbiota.

Sample Type	Groups	Analytical Approach	Key Microbial Changes (Opioids vs. Control)	Functional Implications	Alpha Diversity	Beta Diversity	Key Taxa Modulated	Microbiota-Host Interaction	Statistical Approach	Limitations	Study
Mouse fecal samples	Morphine/fentanyl vs. placebo	Whole genome shotgun sequencing	↓ Firmicutes<br>↑ Akkermansia muciniphila<br>↓ Bifidobacterium pseudolongum<br>↓ Firmicutes/Bacteroidetes ratio	↓ Reg3γ<br>↑ intestinal permeability<br>Barrier disruption<br>Bacterial translocation	↑ Richness (Chao1)	Distinct clustering (PCoA, Bray–Curtis)	↑ Akkermansia muciniphila<br>↓ Bifidobacterium<br>↓ Firmicutes	Opioid receptor-mediated barrier dysfunction	ANOVA; Tukey; PERMANOVA	Male-only cohort<br>No sex-stratified analysis	[39]
Mouse fecal samples	GFR vs. WT (oxycodone-treated)	16S rRNA sequencing	↑ Corynebacterium<br>↑ Staphylococcus<br>↑ Paenibacillus<br>↓ normal murine microbiota	Reduced reward-seeking behavior	↓ Diversity (GFR)	Distinct clustering	↑ Corynebacterium<br>↑ Staphylococcus	Microbiome modulates reward circuitry	ANOVA; FDR correction	Female-only mice<br>No histological validation	[21]
Feces (mice and humans)	Morphine-CPP vs. control; opioid users vs. healthy	16S rRNA sequencing	Mice: ↑ Firmicutes, ↓ Bacteroidetes, ↓ Lactobacillus<br>Humans: ↑ Actinobacteria, ↓ Faecalibacterium	Dysbiosis linked to addiction and mental health	↓ Richness	Significant clustering (PCoA)	Mice: ↑ Firmicutes<br>Humans: ↓ Faecalibacterium	Altered SCFAs; gut–brain axis disruption	Mann–Whitney; ANOVA; LEfSe	Small human cohort<br>Limited opioid diversity	[54]
Rat fecal samples	Morphine vs. sucrose control	16S rRNA sequencing	↓ Firmicutes<br>↓ Clostridium<br>↓ Ruminococcus<br>↑ Bifidobacterium	Reduced SCFAs<br>Neuroinflammation<br>Brain structural changes	↓ Chao1; ↓ Shannon	Distinct clustering (PCoA)	↓ Firmicutes<br>↑ Bifidobacterium	Linked to striatal/thalamic alterations	ANOVA; FDR correction	Antibiotic confounding<br>Subchronic exposure only	[4]
Rat fecal samples	Control vs. fentanyl-dependent	16S rRNA sequencing; MALDI-TOF	↓ microbiome density<br>↓ diversity<br>↓ Ligilactobacillus<br>↓ Escherichia coli	Oxidative stress (MDA ↑)<br>↓ CAT/SOD<br>Behavioral impairment	↓ Shannon; ↓ Chao1	Significant clustering (PCoA)	↓ Ligilactobacillus<br>↓ E. coli	Gut dysbiosis linked to oxidative stress	ANOVA; Tukey	Animal model only<br>Fentanyl-specific	[3]

Table S5: Cocaine-associated alterations in gut microbiota.

Sample Type	Groups	Analytical Approach	Key Microbial Changes (Cocaine vs. Control)	Functional Implications	Alpha Diversity	Beta Diversity	Key Taxa Modulated	Microbiota-Host Interaction	Statistical Approach	Limitations	Study
Fecal pellets	ACQ vs. FACQ (cocaine self-administration)	16S rRNA sequencing; PICRUSt2; mWGS	↑ Barnesiella<br>↑ Ruminococcus<br>↑ Robinsoniella<br>↓ Clostridium IV	Glutamate metabolism linked to cocaine intake	No change	Significant differences (PERMANOVA, p = 0.035)	↓ Lactobacillus (males)<br>↓ Blautia (females)	Gut-brain modules linked to glutamate signaling	GLM; LASSO; PERMANOVA	Mouse model<br>No causal validation	[46]
Fecal samples	Vehicle+cocaine vs. vehicle+saline	16S rRNA sequencing (V3–V4)	↑ Actinobacteria<br>↓ Bacteroides acidifaciens<br>↑ Oscillospira	Persistent dysbiosis after cocaine cessation	↑ Shannon; ↑ richness	Altered beta diversity (Bray–Curtis, UniFrac)	↑ Actinobacteria<br>↓ Bacteroides	CBD modulates cocaine-induced microbiota changes	Kruskal–Wallis; PERMANOVA	Male-only<br>No metagenomics	[8]
Fecal and saliva samples	CUD vs. healthy controls	16S rRNA sequencing; PICRUSt2; GC–MS	↓ Butyrate<br>↑ Erysipelotrichaceae<br>↑ Blautia<br>↓ Christensenellaceae<br>↓ Lachnospiraceae	Inflammation<br>Altered neurotransmitter synthesis<br>SCFA depletion	↓ Alpha diversity	Significant separation (PERMANOVA, p ¡ 0.0001)	↑ Streptococcus (oral)<br>↓ Haemophilus (oral)	Gut–brain axis disruption	PERMANOVA; DESeq2; Mann–Whitney	High dropout<br>Diet not controlled	[16]
Cecal samples	Control vs. antibiotic-treated	16S rRNA sequencing; SCFA metabolomics	↓ SCFAs<br>↑ Proteobacteria<br>↓ Firmicutes	Altered cocaine motivation<br>NAc transcriptional changes	↓ Alpha diversity	Distinct clustering (UniFrac)	↑ Proteobacteria<br>↓ Firmicutes	SCFA depletion alters reward behavior	ANOVA; Tukey; WGCNA	Antibiotic confounding<br>Food restriction bias	[37]
Fecal samples (mice)	Control vs. cocaine (20 mg/kg)	16S rRNA sequencing; PICRUSt2	↑ Akkermansiaceae<br>↓ Muribaculaceae<br>↓ Deferribacteraceae<br>↓ Desulfovibrionaceae<br>Mixed Firmicutes changes	Altered nucleotide metabolism<br>Barrier disruption (mucin degradation)<br>Reduced SCFA producers	No change	Significant separation (Bray–Curtis, Jaccard)	↑ Akkermansia muciniphila<br>↑ Lactobacillus<br>↓ Muribaculum	Barrier dysfunction via mucin degradation	ANOVA; LEfSe; PERMANOVA	No human validation<br>Non-contingent dosing	[2]

Table S6: Metamphetamine-associated alterations in gut microbiota.

Sample Type	Groups	Analytical Approach	Key Microbial Changes (METH vs. Control)	Functional Implications	Alpha Diversity	Beta Diversity	Key Taxa Modulated	Microbiota-Host Interaction	Statistical Approach	Limitations	Study
Fecal samples (mice)	Control vs. METH-exposed mice	16S rRNA sequencing	↑ Lactobacillus<br>↑ Bifidobacterium<br>↓ Bacteroides<br>↓ Muribaculaceae<br>↓ Alistipes	Cardiotoxicity via apoptosis pathways (p53, PI3K/Akt)	↑ Shannon; ↑ Chao1	Distinct clustering (PCoA)	↑ Firmicutes/Bacteroidota ratio<br>↓ Bacteroidota and related taxa	FMT transfers cardiotoxic phenotype	t-test; ANOVA	Short-term model<br>Causality unclear	[31]
Fecal samples (mice)	Control vs. METH-exposed mice	Metagenomic sequencing	No major taxonomic shifts<br>↑ TMAO precursors (choline, L-carnitine)	TMAO-driven neuroinflammation<br>Depression-like behavior	N/A	No significant differences	No consistent taxa changes	TLR4/NF-κB activation (hippocampus)	Wilcoxon; ANOVA	Environmental confounders<br>Unclear TMAO origin	[51]
Fecal samples (Humans)	Healthy controls vs. METH users	16S rRNA sequencing	↓ Bacteroides<br>↓ Roseburia<br>↑ Escherichia-Shigella<br>↑ Bifidobacterium	Oxidative stress<br>Neuropsychiatric symptoms	↓ richness and diversity	Significant separation (Adonis)	↓ SCFA producers<br>↑ opportunistic taxa	Correlation with anxiety/depression scales	Wilcoxon; Pearson correlation	Small sample<br>No dietary control	[53]
Fecal samples (mice)	Prenatal METH vs. control offspring	16S rRNA sequencing	↓ Bacteroidetes<br>↑ Firmicutes<br>↑ Verrucomicrobia<br>↓ Actinobacteria	Oxidative stress<br>Hepatotoxicity<br>Cholestasis	↓ ACE; ↓ Simpson	Distinct clustering (PCoA)	↑ Lactobacillus<br>↑ Saccharimonas<br>↓ Allobaculum	Linked to liver dysfunction markers	ANOVA; Spearman	Prenatal model only<br>No human validation	[25]
Fecal samples (mice)	Saline vs. METH	16S rRNA sequencing	↑ Lachnospiraceae<br>↓ Muribaculaceae	Barrier dysfunction<br>Hormonal dysregulation<br>Oxidative stress	↓ Shannon; ↓ Simpson	Distinct clustering (PCoA)	↑ Lachnospiraceae<br>↓ Bacteroidales	LPS translocation; PI3K–Akt activation	ANOVA; t-test; Spearman	Small sample<br>Short exposure	[29]
Rat fecal samples	Saline vs. METH	16S rRNA sequencing	↑ Klebsiella oxytoca<br>↓ Bacteroidales	Microglial activation<br>Reduced dopamine receptor expression	No change (Shannon)	Distinct clustering (PCoA)	↑ Enterobacteriaceae<br>↓ Porphyromonadaceae	LPS-driven neuroinflammation	ANOVA; t-test; Mann–Whitney	Male-only<br>Antibiotic confounding	[58]
Fecal samples	METH vs. saline	16S rRNA sequencing; GC–MS (SCFAs)	↑ Firmicutes<br>↑ Deferribacterota<br>↓ Bacteroidetes<br>↓ Proteobacteria	Colonic inflammation (TLR4/NF-κB)<br>Barrier disruption<br>↓ SCFAs	↑ richness; no diversity change	Distinct clustering (PCoA)	↑ Lactobacillus<br>↑ Alistipes<br>↓ Muribaculaceae	SCFAs linked to BDNF/TRKB signaling	Kruskal–Wallis; ANOVA; Spearman	Animal model<br>Mechanisms not fully resolved	[59]
Human fecal samples	MA abstainers vs. healthy controls	16S rRNA sequencing	↑ Escherichia-Shigella<br>↑ Enterobacter<br>↓ Bacteroides<br>↓ Bifidobacterium	Barrier disruption<br>Neuroinflammation<br>Impulsivity	↓ diversity indices	Significant separation (PERMANOVA)	↑ Firmicutes<br>↓ Actinobacteria	Microbiota linked to impulsivity behavior	Mann–Whitney; LEfSe; PERMANOVA	Small sample<br>Diet/smoking confounders	[30]
Fecal samples (rats/monkeys)	Pre-MA vs. MA self-administration	16S rRNA sequencing	↓ Clostridia vadin BB60<br>↑ Lactobacillus (rats)	Microbiota linked to MA intake behavior	Rats: ↑ diversity; Monkeys: NS	Rats: significant; Monkeys: NS	↑ Desulfobacteria<br>↓ Proteobacteria	Lactobacillus inversely correlates with intake	PERMANOVA; correlation	Small primate sample<br>No healthy controls	[19]
Human fecal samples	MA users (good sleep) vs. healthy	16S rRNA sequencing	↑ Escherichia-Shigella<br>↑ Faecalitalea<br>↓ Bifidobacterium<br>↓ Bacteroides	Inflammation; reduced probiotics	No change	Significant separation (PERMANOVA)	↑ Firmicutes<br>↓ Actinobacteria	Sutterella linked to sleep dysfunction	Mann–Whitney; LEfSe; Spearman	Cross-sectional<br>Self-reported sleep	[11]

Table S7: Characteristics of included studies evaluating Alcohol abuse and microbiota.

**Place of Study**	**Population**	**Sample Size (n)**	**Age**	**Sex/Gender (% male)**	**SUD Severity**	**Study**
Bethesda, Maryland, USA (NIH Clinical Center)	Treatment-seeking AUD patients	16 patients (no controls)	45 ± 11.6 years	62.5% male	• 17.16 ± 9.78 drinks/day (90 days pre-admission)<br>• ADS score: 21.60 ± 4.21<br>• AUDIT total: 30.27 ± 4.85	[35]
Santiago, Chile (Universidad de Chile)	Genetically selected high-alcohol drinking rats	6 per group (UChB-Alcohol, UChB-Water, Wistar)	2 months old at start	100% male (in experiment)	• 10–12 g ethanol/kg/day<br>• 120 days voluntary consumption	[12]
Wenzhou, Zhejiang, China	Chronic ethanol exposure model	9 per group (Control, Ethanol, E+A, E+L)	3 months old (adult)	100% male	14-week free-choice ethanol paradigm (2%–15% v/v)	[41]
Beijing, China	Alcohol-associated liver disease model	Control: 8<br>Ethanol: 12<br>LI: 8<br>HKLJ: 8	8–10 weeks old	100% male	Lieber-DeCarli diet (4.5%–5.6% ethanol) for 8 weeks	[57]
Hangzhou, China	Ethanol-fed (ALD model)	Not specified*	6–8 weeks old	100% female	Lieber-DeCarli diet (5% ethanol, chronic-binge)	[33]
Zhengzhou University, China	Chronic intermittent alcohol exposure (CIAE) model	16/group (total n=32)	Not specified (6–8 weeks old)	100% male	10% ethanol (v/v) as sole liquid source; stable intake (9 g/kg/12h for 28 days)	[60]
UConn Health, Farmington, CT, USA	AUD patients (DSM-5 diagnosis)	32 (baseline)	Median: 56 (range: 23–70)	47% male / 53% female	• Baseline: 38 ± 18 drinks/week<br>• Endpoint: 17 ± 13 drinks/week (16-week trial)	[10]
Aurora, Colorado, USA	Burn patients with recent alcohol use	19 (12 Low PEth / 7 High PEth)	47.7 ± 14.8 years	89.5% male / 10.5% female	High PEth (≥20 ng/mL) indicates recent heavy drinking; AUDIT-C confirmed AUD	[20]
Shandong, China (Liaocheng Univ.)	Ethanol-induced liver injury model	16 (8 Ethanol vs. 8 Control)	6–8 weeks old (young adults)	100% male	Binge ethanol (5.5 g/kg BW, 3 doses)	[50]
Taiwan, New Taipei City	Alcohol-induced liver disease (ALD) model	24 rats (8 per group)	6-week-old males	100% male	Chronic ethanol feeding (10.4 g/kg BW/day)	[7]
China, Heilongjiang, Qiqihar	Alcohol-withdrawn depressive model	30 rats (6 per group)	200 ± 20 g (weight)	100% male	2 g/kg/day for 4 weeks, followed by withdrawal	[26]
China, Dalian	Alcohol-induced liver injury model	7 per group (NG, MG, SLF)	7 weeks old	100% male	Chronic alcohol intake (6–12 mL/kg/day)	[47]
USA, Colorado, Aurora	Aged and young mice with ethanol exposure	4–11 per group (young/aged, ±ethanol)	Young: 4–5 months; Aged: 21–22 months	100% female	Moderate ethanol exposure (1.5 g/kg and 1.25 g/kg)	[36]
China, Xinxiang City	Alcohol-dependent (AD) patients	34 AD / 19 controls	39.24 ± 1.72 (AD), 39.58 ± 2.76 (controls)	100% male	Alcohol intake: 489.42 ± 29.91 g/day (AD), 8.68 ± 2.22 g/day (controls)<br>AUDIT score: 33.59 ± 0.39 (AD), 1.47 ± 0.50 (controls)	[50]

Table S8: Characteristics of included studies evaluating alcohol abuse and microbiota.

Place of Study	Population	Sample Size (n)	Age	Sex/Gender (% male)	SUD Severity	Study
Wuhan, China	MetALD model (HFHSAD diet)	8/group	150–180 g (BW)	100% male	High-fat/high-sucrose + 22% ethanol	[31]
China (Xi'an, Shenzhen)	Pubertal and adult mice exposed to alcohol	20 mice (pubertal and adult groups)	Pubertal: 3 weeks; Adult: 7 weeks	100% male	Chronic alcohol exposure (20% v/v for 2 weeks)	[55]
Wuxi, China	Ethanol-fed mice	Not specified	8 weeks old	100% male	5% ethanol liquid diet (21 days)	[40]
Xinxiang, China	Binge-on-chronic ethanol	24 (8–9/group)	8–10 weeks old	100% male	5% ethanol diet + 4 g/kg binge gavage	[43]
Suzhou, China	HFD + ethanol co-exposure	7/group	6–8 weeks old	Not specified	20% ethanol (v/v) + 60% fat diet	[51]
Nanjing, China	Acute alcoholic liver injury	6/group	6 weeks old	100% male	50% ethanol (12 mL/kg body weight)	[44]
Greece (Athens)	AUD inpatients	20 (14 responders, 6 non-responders)	52 ± 6.93 years	85% male	DSM-5 diagnosis; detoxification program	[24]
Finland (FinnGen cohort)	AUD population (registry-based)	169,244 (2,660 cases)	N/A	N/A	Registry-based AUD diagnosis	[52]

Table S9: Characteristics of included studies evaluating Opioid abuse and microbiota.

**Place of Study**	**Population**	**Sample Size (n)**	**Age**	**Sex/Gender (% male)**	**SUD Severity**	**Study**
USA (Boston, MA)	Female GFR vs. WT mice	GFR: 20; WT: 20	7–8 weeks	100% female	Oxycodone CPP model	[21]
China (Shijiazhuang)	Morphine-CPP mice; Opioid addicts	Mice: 64<br>Humans: 58 opioid addicts, 50 controls	Mice: 7–8 weeks<br>Humans: Median 39.5 years	Humans: Not specified	Morphine-CPP model<br>DSM-5 criteria (humans)	[54]
USA (Fort Worth, Texas)	Morphine self-administering rats	13 males, 13 females	Adult (not specified)	50% male, 50% female	14 days morphine IV self-administration (0.4 mg/kg/infusion)	[4]
Marrakech, Morocco	Fentanyl-dependent rats	6 per group (total n = 30)	18–19 weeks old	100% male	Chronic fentanyl (30 days)<br>Escalating doses	[3]
Richmond, Virginia, USA	Opioid-dependent (morphine/fentanyl)	Not specified*	6–8 weeks old	100% male	Chronic morphine pellets<br>or escalating fentanyl doses	[39]

Table S10: Characteristics of included studies evaluating multiple SUD and microbiota.

**Place of Study**	**Population**	**Sample Size (n)**	**Age**	**Sex/Gender (% male)**	**SUD Severity**	**Study**
Shantou, China	Ketamine-addicted rats	7 KET / 7 SAL	6–8 weeks	100% male	CPP model (30 mg/kg ketamine)	[54]
Shanghai / Yunnan / Shandong, China	SUD (methamphetamine/opioids)	333 SUD / 143 non-SUD	Not reported	Not reported	DSM-5 criteria Multiple arrests	[32]
Guangzhou, China	FLZ-exposed vs. control (zebrafish)	30 fish/group	5 months old	Not specified	0.2 μg/L and 5 μg/L FLZ 30 days exposure	[27]

Table S11: Characteristics of included studies evaluating cocaine use and microbiota.

Place of Study	Population	Sample Size (n)	Age	Sex/Gender (% male)	SUD Severity	Study
Farmington, CT, USA	Cocaine IVSA (ACQ vs. FACQ)	228 total<br>ACQ: 128; FACQ: 100	3–6 months	M: 53%, F: 47%	Acquisition of cocaine self-administration	[46]
Campbelltown, NSW, Australia	Cocaine CPP (CBD vs. Vehicle)	48 total<br>VEH-COC: 12; CBD-COC: 13	8 weeks at arrival	100% male	7 × 20 mg/kg cocaine conditioning	[8]
Florence, Italy	Cocaine Use Disorder (CUD) patients	58 CUD / 20 HC	40.4 years (range 20–58)	83% male / 17% female	29% daily use<br>62% ¿10 years use	[16]
New York, USA	Cocaine Use Disorder (CUD) model	Experiment 1:<br>10 H_2O / 7 Abx	Adult (300–320 g)	100% male	Self-administered cocaine (0.8 mg/kg/infusion)	[37]
USA (Detroit, MI)	Cocaine-treated vs. saline controls	6–7 mice/group	Young adults (not specified)	100% female	10-day cocaine HCl (20 mg/kg, IP)	[2]

Table S12: Characteristics of included studies evaluating methamphetamine use and microbiota.

Place of Study	Population	Sample Size (n)	Age	Sex/Gender (% male)	SUD Severity	Study
Guangzhou, China	METH-exposed mice	8/group	6–8 weeks	100% male	Escalating doses (1.5–10 mg/kg)	[31]
Shijiazhuang, China	METH-exposed mice	6–20/group	8 weeks	100% male	Binge doses (10 mg/kg, 4 injections)	[51]
China (Shanxi and Hebei Provinces)	Methamphetamine abusers with mental disorders	223 (139 METH, 84 HCs)	19–56 years	65.9% female / 34.1% male	Frequency: once/day to 1–3 times/month<br>Duration: 1–6 days to ≥1 year	[53]
China (Guangzhou)	Prenatal methamphetamine-exposed offspring	6 pregnant mice/group (offspring analyzed)	PND42	Equal sex distribution	5 mg/kg/day during pregnancy (PD0.5–PD17.5)	[25]
Guangzhou, China	Methamphetamine-exposed mice	20 total (Saline: 10; METH: 10)	8–10 weeks	100% male	Escalating dose–multiple binge model	[29]
Beijing, China	Methamphetamine self-administration	25 total (Saline: 10; METH: 15)	260–280 g (adult)	100% male	Self-administration (0.1 mg/kg/infusion)	[58]
Guangzhou, China (Southern Medical University)	Methamphetamine-administered mice	Meth: ∼10<br>Saline: ∼10	8–10 weeks old	Not specified (likely male)	15 mg/kg Meth 4-day regimen (human-equivalent exposure)	[59]
China, Shenyang, Liaoning	Methamphetamine use disorder (MUD)	62 MA / 50 HC	MA: 41.00 (34.00, 50.00) yrs<br>HC: 43.50 (35.00, 53.00) yrs	MA: 67.7% male; HC: 60% male	Abstinence ¡3 months (baseline)<br>Follow-up: 2 months	[30]
China, Changsha, Hunan	Methamphetamine self-administration (rats/monkeys)	Rats: 10 MA<br>Monkeys: 4 MA	Rats: 220–250 g<br>Monkeys: 9–12 years	100% male	Rats: 0.05 mg/kg/infusion<br>Monkeys: 0.032–1 mg/kg (PR)	[19]
China, Shenyang, Liaoning	Methamphetamine use disorder (MUD)	70 MA (49 MA-GS, 21 MA-BS)<br>38 HC	MA-GS: 38.98 ± 9.65 yrs<br>HC: 42.74 ± 9.38 yrs	MA: 71.4% male; HC: 60.5% male	Abstinence: 2–6 months<br>DSM-5 criteria	[11]

Table S13: Risk of bias assessment of included animal studies

Study	Demographic diff.	Habitat stability	Genotype/ familial/ source	Extreme diet	GMB norm.	Extreme genotype	Randomiz- ation	Intervention bias	Validation method	Deviation/ interv.	Missing data	Subject dropout	Sequencing depth/ zeros	Sample collec- tion	Blinding	Selection reported results	Overall
[12]	Low	Low	Low	Low	Low	Low	Low	Low	Low	Low	Low	Low	Low	Low	N/A	Low	**Low**
[41]	Low	Low	Low	Low	Low	Low	Low	Low	Low	Low	Low	Low	Low	Low	N/A	Low	**Low**
[57]	Low	Low	Low	Low	Low	Low	Low	Low	Low	Low	Low	High	Low	Low	N/A	Low	**High**
[33]	Low	Low	Low	Low	Low	Low	Low	Low	Low	Low	Low	Low	Low	Low	N/A	Low	**Low**
[39]	Low	Low	Low	Medium	Low	Low	Low	Low	Low	Low	Low	Low	Low	Low	High	Low	**High**
[60]	Low	Low	Low	Low	Low	Low	Low	Low	Low	Low	Low	Low	Low	Low	Low	Low	**Low**
[50]	Low	Low	Low	Low	Low	Low	Low	Low	Low	Low	Low	Low	Low	Low	Low	Low	**Low**
[7]	Low	Low	Low	Low	Low	Low	Low	Low	Low	Low	Low	Low	Low	Low	Low	Low	**Low**
[25]	Low	Low	Low	Low	Low	Low	Low	Low	Low	Low	Low	Low	Low	Low	Low	Low	**Low**
[36]	Low	Low	Low	Low	Low	Low	Low	Low	Low	Low	Low	Low	Low	Low	Low	Low	**Low**
[53]	Low	Low	Low	Medium	Low	Low	Low	Low	Low	Low	Low	Low	Low	Low	Low	Low	**Medium**
[55]	Low	Low	Low	Low	Low	Low	Low	Low	Low	Low	Low	Low	Low	Low	Low	Low	**Low**
[40]	Low	Low	Low	Low	Low	Low	Low	Low	Low	Low	Low	Low	Low	Low	Low	Low	**Low**
[43]	Low	Low	Low	Low	Low	Low	Low	Low	Low	Low	Low	Low	Low	Low	Low	Low	**Low**
[51]	Low	Low	Low	Low	Low	Low	Low	Low	Low	Low	Low	Low	Low	Low	High	Low	**High**
[44]	Low	Low	Low	Low	Low	Low	Low	Low	Low	Low	Low	Low	Low	Low	Low	Low	**Low**
[54]	Low	High	Low	High	High	Low	Low	Low	Low	Low	Low	Low	Low	Low	Low	Low	**High**
[27]	Low	Low	Low	Low	Low	Low	Low	Low	High	Low	Low	Low	Low	Low	Low	Low	**High**
[49]	Low	Low	Low	Medium	High	Low	High	Low	Low	Low	Low	Low	Low	Low	Low	Low	**High**
[13]	Low	Low	Low	Medium	Low	Low	Low	Low	Low	Low	Low	Low	Low	Low	Low	Low	**Medium**
[56]	Low	Low	Low	Medium	Low	Low	Low	Low	Low	Low	Low	Low	Low	Low	Low	Low	**Medium**
[21]	Low	Low	Low	Medium	Low	Low	Low	Low	Low	Low	Low	Low	Low	Low	Low	Low	**Medium**
[4]	Low	Low	Low	Low	Low	Low	Low	Low	Low	Low	Low	Low	Low	Low	Low	Low	**Low**
[3]	Low	Low	Low	Medium	Low	Low	Low	Low	Low	Low	Low	Low	Low	Low	Low	Low	**Medium**
[27]	Low	Low	Low	Low	Low	Low	Low	Low	Low	Low	Low	Medium	Low	Low	Low	Low	**Medium**
[46]	Low	Low	Low	Low	Low	Low	Low	Low	Low	Low	Low	Low	Low	Low	Low	Low	**Low**
[8]	Low	Low	Low	Medium	Low	Low	Low	Low	Low	Low	Low	Low	Low	Low	Low	Low	**Medium**
[37]	Low	Low	Low	Low	Low	Low	Low	Low	Low	Low	Low	Low	Low	Low	Low	Low	**Low**
[2]	Low	Low	Low	Low	Low	Low	Low	Low	Low	Low	Low	Low	Low	Low	Low	Low	**Low**
[31]	Low	Low	Low	Low	Low	Low	Low	Low	Low	Low	Low	Low	Low	Low	Low	Low	**Low**
[48]	Low	Low	Low	Medium	Low	Low	Low	Low	Low	Low	Low	Low	Low	Low	High	Low	**High**
[29]	Low	Medium	Low	Low	Low	Low	Low	Low	Low	Low	Low	Low	Low	Low	Low	Low	**Medium**
[30]	Low	Low	Low	Low	Low	Low	Low	Low	Low	Low	Low	Low	Low	Low	Low	Low	**Low**
[58]	Low	Low	Low	Low	Low	Low	Low	Low	Low	Low	Low	Low	Low	Low	Low	Low	**Low**
[59]	Low	Low	Low	Low	Low	Low	Low	Low	Low	Low	Low	Low	Low	Low	Low	Low	**Low**
[6]	Low	Low	Low	Low	Low	Low	Low	Low	Low	Low	Low	Low	Low	Low	Low	Low	**Low**
[28]	Low	Low	Low	Low	Low	Low	Low	Low	Low	Low	Low	Low	Low	Low	Low	Low	**Low**
[19]	Low	Low	Low	Low	Low	Low	Low	Low	Low	Low	Low	Low	Low	Low	Low	Low	**Low**

Score key: Low = Low risk of bias; Medium = Moderate risk of bias; High = High risk of bias; N/A = Not applicable.

Table S14: Risk of bias assessment of included human studies.

Study	Demographic diff.	Habitat stability	Genotype/ familial/ source	Extreme diet	GMB norm.	Extreme genotype	Randomiz- ation	Intervention bias	Validation method	Deviation/ interv.	Missing data	Subject dropout	Sequencing depth/ zeros	Sample collec- tion	Blinding	Selection reported results	Overall
[35]	High	Low	Medium	Medium	Low	Low	High	Low	Low	Low	Low	Low	Low	Low	High	Low	**High**
[10]	Low	Medium	Low	High	Low	High	Low	Low	Low	Low	High	High	Low	Low	Low	Low	**High**
[20]	Low	Medium	High	High	Low	High	High	Low	Low	Low	Low	Low	Low	Low	High	Low	**High**
[28]	Medium	Low	High	High	Low	High	High	Low	N/A	N/A	Low	Medium	Low	Low	High	Low	**High**
[24]	High	High	Low	High	High	High	High	Low	Low	Low	Low	Medium	Low	Low	High	Low	**High**
[49]	N/A	N/A	N/A	N/A	N/A	N/A	Low	N/A	N/A	N/A	N/A	N/A	Low	Low	Low	Low	**Low**
[32]	Medium	N/A	High	High	High	High	High	N/A	N/A	Low	Low	Low	Low	Low	Low	Low	**High**
[16]	Low	N/A	Medium	N/A	High	N/A	Low	Low	Low	Low	Low	Low	Low	Low	Low	Low	**High**
[50]	Low	N/A	N/A	High	N/A	High	Low	Low	Low	Low	High	High	Low	Low	High	Low	**High**
[11]	Low	N/A	Medium	High	High	High	Low	Low	Low	Low	Low	Low	Low	Low	Low	Low	**High**

Score key: Low = Low risk of bias; Medium = Moderate risk of bias; High = High risk of bias; N/A = Not applicable.

PRISMA 2020 Checklist

Section and Topic	Item #	Checklist item	Location where item is reported
**TITLE**			
Title	1	Identify the report as a systematic review.	Line 1-4
**ABSTRACT**			
Abstract	2	See the PRISMA 2020 for Abstracts checklist.	Abstract lines 15–38
**INTRODUCTION**			
Rationale	3	Describe the rationale for the review in the context of existing knowledge.	Lines 77–91
Objectives	4	Provide an explicit statement of the objective(s) or question(s) the review addresses.	Lines 79–91
**METHODS**			
Eligibility criteria	5	Specify the inclusion and exclusion criteria for the review and how studies were grouped for the syntheses.	Lines 109–120
Information sources	6	Specify all databases, registers, websites, organisations, reference lists and other sources searched or consulted to identify studies. Specify the date when each source was last searched or consulted.	Lines 96–102
Search strategy	7	Present the full search strategies for all databases, registers and websites, including any filters and limits used.	Lines 103–108
Selection process	8	Specify the methods used to decide whether a study met the inclusion criteria of the review, including how many reviewers screened each record and each report retrieved, whether they worked independently, and if applicable, details of automation tools used in the process.	Lines 125–128
Data collection process	9	Specify the methods used to collect data from reports, including how many reviewers collected data from each report, whether they worked independently, any processes for obtaining or confirming data from study investigators, and if applicable, details of automation tools used in the process.	Lines 134–139
Data items	10a	List and define all outcomes for which data were sought. Specify whether all results that were compatible with each outcome domain in each study were sought (e.g. for all measures, time points, analyses), and if not, the methods used to decide which results to collect.	Lines 134–149
	10b	List and define all other variables for which data were sought (e.g. participant and intervention characteristics, funding sources). Describe any assumptions made about any missing or unclear information.	Lines 134–149
Study risk of bias assessment	11	Specify the methods used to assess risk of bias in the included studies, including details of the tool(s) used, how many reviewers assessed each study and whether they worked independently, and if applicable, details of automation tools used in the process.	Lines 129–133 and Tables 13-14 of supplementary materials
Effect measures	12	Specify for each outcome the effect measure(s) (e.g. risk ratio, mean difference) used in the synthesis or presentation of results.	Lines 149–152
Synthesis methods	13a	Describe the processes used to decide which studies were eligible for each synthesis (e.g. tabulating the study intervention characteristics and comparing against the planned groups for each synthesis (item #5)).	Lines 147–152
	13b	Describe any methods required to prepare the data for presentation or synthesis, such as handling of missing summary statistics, or data conversions.	Lines 134–139
	13c	Describe any methods used to tabulate or visually display results of individual studies and syntheses.	Results sections (3.1–3.6); ROBINS-I stacked bar charts (Figure 2); Supplementary

Section and Topic	Item #	Checklist item	Location where item is reported
			Tables 1–14
	13d	Describe any methods used to synthesize results and provide a rationale for the choice(s). If meta-analysis was performed, describe the model(s), method(s) to identify the presence and extent of statistical heterogeneity, and software package(s) used.	Lines 139 –146
	13e	Describe any methods used to explore possible causes of heterogeneity among study results (e.g. subgroup analysis, meta-regression).	Lines 139–146
	13f	Describe any sensitivity analyses conducted to assess robustness of the synthesized results.	no sensitivity analyses were conducted
Reporting bias assessment	14	Describe any methods used to assess risk of bias due to missing results in a synthesis (arising from reporting biases).	Lines 129-136<br>Figure 2<br>Supplementary material: Table 13-14
Certainty assessment	15	Describe any methods used to assess certainty (or confidence) in the body of evidence for an outcome.	Lines 152–164
**RESULTS**			
Study selection	16a	Describe the results of the search and selection process, from the number of records identified in the search to the number of studies included in the review, ideally using a flow diagram.	Methods/Results (lines 118–121)
	16b	Cite studies that might appear to meet the inclusion criteria, but which were excluded, and explain why they were excluded.	Figure 1
Study characteristics	17	Cite each included study and present its characteristics.	Supplementary Tables 7–12
Risk of bias in studies	18	Present assessments of risk of bias for each included study.	Lines 166–173,<br>Supplementary material: tables 13-14
Results of individual studies	19	For all outcomes, present, for each study: (a) summary statistics for each group (where appropriate) and (b) an effect estimate and its precision (e.g. confidence/credible interval), ideally using structured tables or plots.	Results sections 3.1–3.6
Results of syntheses	20a	For each synthesis, briefly summarise the characteristics and risk of bias among contributing studies.	Lines 392–399 , Figure 2, Supplementary material: tables 13-14
	20b	Present results of all statistical syntheses conducted. If meta-analysis was done, present for each the summary estimate and its precision (e.g. confidence/credible interval) and measures of statistical heterogeneity. If comparing groups, describe the direction of the effect.	Not applicable
	20c	Present results of all investigations of possible causes of heterogeneity among study results.	Not applicable
	20d	Present results of all sensitivity analyses conducted to assess the robustness of the synthesized results.	Not applicable
Reporting biases	21	Present assessments of risk of bias due to missing results (arising from reporting biases) for each synthesis assessed.	Lines 129-134<br>Figure 2<br>Supplementary

Section and Topic	Item #	Checklist item	Location where item is reported
			material: Table 13-14
Certainty of evidence	22	Present assessments of certainty (or confidence) in the body of evidence for each outcome assessed.	Lines 156–164
**DISCUSSION**			
Discussion	23a	Provide a general interpretation of the results in the context of other evidence.	Lines 384–516
	23b	Discuss any limitations of the evidence included in the review.	Lines 492–499
	23c	Discuss any limitations of the review processes used.	Discussion (lines 492–499) + Methods (lines 152–156)
	23d	Discuss implications of the results for practice, policy, and future research.	Lines 498–516
**OTHER INFORMATION**			
Registration and protocol	24a	Provide registration information for the review, including register name and registration number, or state that the review was not registered.	Lines 152–156
	24b	Indicate where the review protocol can be accessed, or state that a protocol was not prepared.	Lines 152–156. No protocol available.
	24c	Describe and explain any amendments to information provided at registration or in the protocol.	Not applicable
Support	25	Describe sources of financial or non-financial support for the review, and the role of the funders or sponsors in the review.	Funding section (lines 554–555)
Competing interests	26	Declare any competing interests of review authors.	Line 565
Availability of data, code and other materials	27	Report which of the following are publicly available and where they can be found: template data collection forms; data extracted from included studies; data used for all analyses; analytic code; any other materials used in the review.	Lines 558–559

From: Page MJ, McKenzie JE, Bossuyt PM, Boutron I, Hoffmann TC, Mulrow CD, et al. The PRISMA 2020 statement: an updated guideline for reporting systematic reviews. BMJ 2021;372:n71. doi: 10.1136/bmj.n71.
This work is licensed under CC BY 4.0. To view a copy of this license, visit https://creativecommons.org/licenses/by/4.0/

Figure S1. PRISMA 2020 checklist.

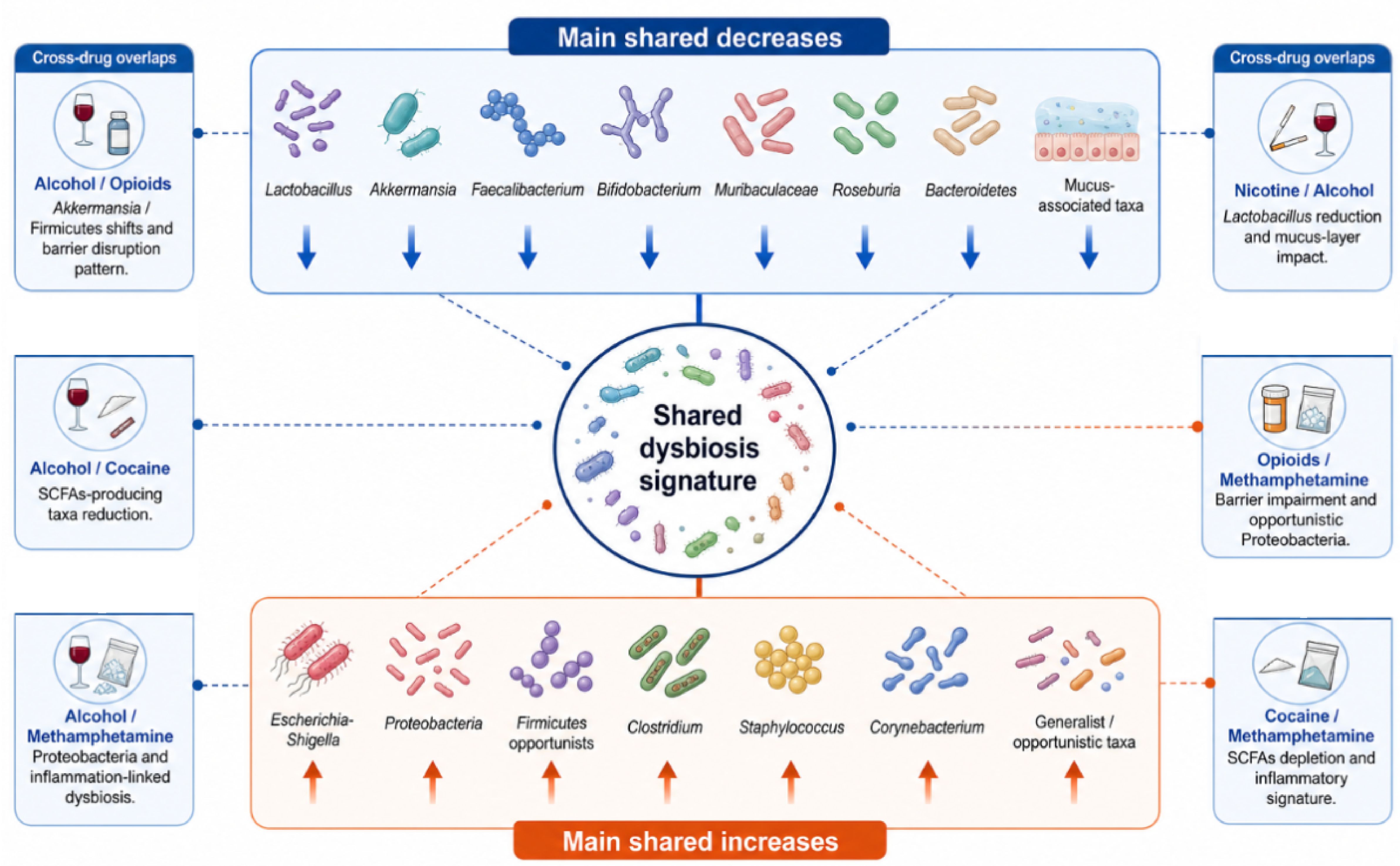

Figure S2: Main taxonomic shifts shared across substances of abuse and specific drugs contributing to their specific patterns.
